# Identification of LACTB2, a metallo-β-lactamase protein, as a human mitochondrial endoribonuclease

**DOI:** 10.1093/nar/gkw050

**Published:** 2016-01-29

**Authors:** Shiri Levy, Charles K. Allerston, Varda Liveanu, Mouna R. Habib, Opher Gileadi, Gadi Schuster

**Affiliations:** 1 Faculty of Biology, Technion- Israel Institute of Technology, Haifa 32000, Israel; 2 Structural Genomics Consortium, Nuffield Department of Medicine, University of Oxford, Oxford OX3 7DQ, UK

## Abstract

Post-transcriptional control of mitochondrial gene expression, including the processing and generation of mature transcripts as well as their degradation, is a key regulatory step in gene expression in human mitochondria. Consequently, identification of the proteins responsible for RNA processing and degradation in this organelle is of great importance. The metallo-β-lactamase (MBL) is a candidate protein family that includes ribo- and deoxyribonucleases. In this study, we discovered a function for LACTB2, an orphan MBL protein found in mammalian mitochondria. Solving its crystal structure revealed almost perfect alignment of the MBL domain with CPSF73, as well as to other ribonucleases of the MBL superfamily. Recombinant human LACTB2 displayed robust endoribonuclease activity on ssRNA with a preference for cleavage after purine-pyrimidine sequences. Mutational analysis identified an extended RNA-binding site. Knockdown of LACTB2 in cultured cells caused a moderate but significant accumulation of many mitochondrial transcripts, and its overexpression led to the opposite effect. Furthermore, manipulation of LACTB2 expression resulted in cellular morphological deformation and cell death. Together, this study discovered that LACTB2 is an endoribonuclease that is involved in the turnover of mitochondrial RNA, and is essential for mitochondrial function in human cells.

## INTRODUCTION

Mitochondria are important for many metabolic pathways, including the production of adenosine triphosphate (ATP) in the process of oxidative phosphorylation. Mitochondria are an evolutionary remnant of an endosymbiotic event that occurred 1.5 billion years ago, originating from the α-proteobacterium, where most of the bacterium genes were transferred from the organelle to the nuclear genome of the ancient host (1,2). The mammalian mitochondrial genome preserved a total of 37 genes encoding two ribosomal RNAs, 22 tRNA genes and 13 proteins encoding oxidative phosphorylation components subunits, such as NADH dehydrogenase, ATP synthase and cytochrome oxidase. These components are essential for cell viability (3,4). Mitochondrial RNAs are transcribed from the mitochondrial DNA as polycistronic molecules, in which the mRNAs and rRNAs are punctuated by tRNAs (3–5). Endonucleolytic cleavages of tRNAs at both the 5΄ and 3΄ ends are performed by RNase P and RNase Z, respectively, generating, in addition to mature tRNAs, processed rRNA and mRNA transcripts (5,6). The released individual RNA species are then decorated with stable poly(A)-tails, and the mRNAs are translated by the mitochondrial ribosomes. Aside from the addition of the stable poly(A)-tail at the 3΄ end, the addition of transient and unstable poly(A)-tails to truncated transcripts has been observed (7). These tails may represent the polyadenylation-assisted degradation pathway of RNA described in bacteria, archaea and organelles, as well as in the nucleus and cytoplasm (8–11). However, it should be noted that despite these strong evidence, the poly(A)-assisted degradation pathway has not yet been proven to take place in human mitochondria. Although produced from few polycistronic transcripts, the rRNA, tRNA and mRNA transcripts accumulate in the mitochondria to different concentrations, indicating the importance of a modulated and well-controlled RNA degradation mechanism. The presence of RNA granules within the mitochondria has been recently described (12–15). These mitochondrial granules are associated with RNA-binding proteins and enzymes that are functionally linked to the processing and degradation of mitochondrial transcripts. Therefore, it is thought that these activities are localized to these novel mitochondrial compartments.

To better understand defects in mitochondrial RNA turnover and, consequently, mitochondrial disorders, extensive investigations are underway to identify the ribonucleases that are responsible for the processing and degradation of mitochondrial transcripts (3,4). The mitochondrial RNase P and RNase Z (ELAC2), which process and punctuate the tRNAs, were previously characterized (6,16,17). The human mitochondrial polynucleotide phosphorylase (PNPase) has been indicated as the natural candidate for the 3΄ to 5΄ exoribonuclease activity on mitochondrial RNA and the transient polyadenylation of RNA, as established for these functions in prokaryotes and organelles (14,18–20). However, this assumption was questioned when PNPase was found to be primarily located in the mitochondrial intermembrane space (21). Nevertheless, a recent work has shown that a significant amount of this protein is present in RNA granules in complex with the hSuv3p helicase (14). This complex, termed the mitochondrial exosome, degrades ‘mirror’ RNAs that are complementary to mitochondrial genes within the RNA granules described above. An additional RNA exonuclease, termed REXO2, is located both in the intermembrane space and the matrix and it has been proposed to degrade oligo-ribonucleotides that are generated by PNPase and other ribonucleases (22). Another known ribonuclease is PDE12, a mitochondrial 2΄- and 3΄-phosphodiesterase that has been shown to removed stable poly(A)-tails *in vitro* and in cultured cells (23). Several RNA-binding proteins that are important for the correct processing and stability of mitochondrial transcripts, but that do not possess ribonucleolytic activity, were also described (12,13,15,16,24–26). Endonuclease G is a powerful non-specific DNA/RNA endonuclease that is located in the intermembrane space of the mitochondria and functions during apoptosis (27,28). Aside from the two tRNA-punctuating enzymes RNase P and RNase Z, no endoribonuclease has been detected in the mitochondrial matrix. Therefore, we sought to identify a candidate for this activity that potentially functions in the degradation of mitochondrial transcripts.

A candidate superfamily of proteins that are responsible for nucleic acid metabolism and which have been observed in all three domains of life is the metallo-β-lactamase (MBL) superfamily. The MBL superfamily includes a variety of proteins that are responsible for RNA processing, DNA repair and small-molecule metabolism (29–31). Functionally, MBL proteins are metallo-enzymes requiring one or two zinc ions for their activity, while using one water/hydroxyl molecule as a ligand and a wide variety of substrates that share ester linkages and a negative charge (29,32). Structurally, most of the 6000 members of this superfamily have a common four-layered αβ/βα conformation and share five conserved sequence motifs in their active site: Asp (I), His-X-His-X-Asp-His (II) (where X indicates any amino acid), His (III), Asp (IV), and His (V); Motif II is referred to as the signature sequence motif of the MBL superfamily (29).

Based on extensive sequence analysis and the prospective biological function of MBL proteins, several subfamilies have been classified, two of which (ELAC and β-CASP) were identified as nucleic acid hydrolases (32,33). The ELAC subfamily includes the RNase Z (ELAC2) endoribonuclease located in the mitochondria (34). The β-CASP subfamily (MBL, CPSF, Artemis, SNM1/PSO2), whose members are key players in RNA metabolism (CPSF-73 and RNase J) and DNA crosslink repair (Artemis and its homologs mouse SNM1 and yeast PSO2), has been studied over the past decade (32,35). RNA nucleases from the β-CASP subfamily are found in all domains of life and encompass distinct activities. The *Bacillus subtilis* RNase J and the *Arabidopsis thaliana* chloroplast RNase J share dual endonucleolytic (endo-) and exonucleolytic (exo-) activities and are catalyzed in the same catalytic vicinity (35–39). The processive exonucleolytic activity is performed in the 5΄ to 3΄ direction, making it the first 5΄ to 3΄ exonuclease discovered in prokaryotes (36). The mammalian CPSF-73 (cleavage and polyadenylation specificity factor) shares similar characteristics, as it is responsible for the endo-cleavage of pre-mRNA 3΄ processing and the dual endo-/exo- 5΄ to 3΄ activity on pre-histone mRNA (31,40,41). Three RNase J/CPSF homologs were characterized as exclusive endo- or exo-ribonucleases in the hyperthermophilic methanogenic archaea *Methanocaldoccous jannaschii* (42).

Prompted by the above findings, we searched for mitochondrial MBL proteins featuring the traditional characteristics of MBL ribonucleases. β-lactamase-like-protein 2 (LACTB2), has been suggested to be located in the mitochondria (43). Other than the bioinformatic prediction that LACTB2 belongs to the glyoxalase II subfamily, no studies regarding its activity and function have been previously described. Being an orphaned protein located in the mitochondria and preserving the prospective ribonucleolytic MBL motifs, we hypothesized that LACTB2 is a ribonuclease that may be involved in the RNA processing and/or degradation in that organelle. We report the characterization of LACTB2 as monomeric soluble protein, exhibiting endoribonuclease activity in human mitochondria, essential for mitochondria functioning and cell viability.

## MATERIALS AND METHODS

### Identification, alignment and structural modeling

Human LACTB2 was first identified in (http://www.mitoproteome.org/) (44,45) and later in UniProt (Q53H82). Selected proteins were aligned using multiple sequences alignment by Clustal W2 and analyzed using JalView. The structural alignments, electrostatic potential surface calculation and molecular graphics were performed using the UCSF Chimera software (https://www.cgl.ucsf.edu/chimera/) (46).

### Cloning, expression and purification of LACTB2

Human LACTB2 cDNA was polymerase chain reaction (PCR) amplified using the appropriate oligonucleotide primers (Supplementary Table S1) and inserted, via ligation-independent cloning, into the bacterial expression vector pNIC28-Bsa4 (47). Six sequential His residues followed by a Tobacco Etch Virus (TEV) protease cleavage site were located on the vector downstream of the T7 promoter, allowing N-terminal fusion with the LACTB2 insert. Since the recombinant protein without the putative mitochondrial transit peptide was insoluble, the protein was expressed with the putative transit peptide. Site-directed mutagenesis was performed using PCR, appropriate oligonucleotide primers (Supplementary Table S2) and PFU or Herculase II (Thermo Scientific) (42). *Escherichia coli* DE3 *pnp*- cells (DEHO: C-5691) (48) were transformed with the LACTB2 WT expression plasmid (or mutants) and grown at 37°C in TB medium to an OD_600_ of 1.0 (42). Protein expression was induced with 0.1 mM IPTG for 16 h at 18°C. Protein for crystallization was expressed in BL21(DE3)-R3-pRARE2 cells (47). Cells were collected by centrifugation and frozen at −80°C. To purify the proteins, bacteria were suspended in four volumes of lysis buffer (50 mM HEPES, pH 7.4, 500 mM NaCl, 5 mM imidazole, 5% glycerol, 0.5 mM TCEP) supplemented with Protease Inhibitor Cocktail Set VII (Calbiochem, 1/1000 dilution). The cell suspension was lysed with a microfluidizer, polyethyleneimine was added to 0.15% (from a 5% w/v, pH 7.5 stock solution) and cell debris and precipitated nucleic acids were removed by centrifugation for 30 min at 25 000 *g*. The soluble fraction was applied to a 1-ml Histrap column (GE Healthcare); the column was washed with lysis buffer containing 25 mM imidazole, then eluted with lysis buffer containing 250 mM imidazole. The eluted fraction was combined with TEV protease (1:20 w/w) and dialyzed overnight at 4°C against buffer A (50 mM HEPES pH7.4, 500 mM NaCl, 5% glycerol, 25 mM imidazole, 0.5 mM TCEP), and reloaded onto a Ni column. The TEV protease and other impurities bound the Ni column, while LACTB2 was collected in the flow-through fraction. The protein was concentrated using Vivaspin Centricon (Satorius) and loaded on a gel filtration Superdex 200 HR 10/300 column using GF buffer (25 mM Tris–HCl, 250 mM NaCl pH 7.4, 5% glycerol and 0.5 mM TCEP). The fraction containing LACTB2, which was ∼30–50 kDa, was either concentrated and used directly for crystallization, or (for activity assays) dialyzed against low-salt Q buffer (25 mM Tris–HCl pH = 7.4, 25 mM NaCl, 5% glycerol and 0.5 mM DTT), passed through a MonoQ column (GE Healthcare) and collected in the flow-through fraction. The purified protein was dialyzed against its 2× activity buffer (25 mM Tris–HCl pH 7.4, 250 mM NaCl, 5% glycerol, 0.5 mM DTT), fast-frozen in liquid nitrogen and stored at −80°C. LACTB2 preparations used for activity assays were assayed both enzymatically and by immunoblot assay to detect any traces of contaminating *E. coli* ribonucleases: RNase E, PNPase, RNase II and RNase R.

### Crystallization

Poorly diffracting crystals (7–11Å) were grown by vapor diffusion at 20°C in sitting drops (50 nl of 15 mg/ml protein, 5 mM dCMP and 100 nl of precipitant consisting of 0.2 NaBr, 0.1M Bis-Tris-Propane, pH 6.5, 20% PEG 3350 and 10% Ethylene Glycol), and improved crystals diffracting to 2.6 Å were obtained by seeding.

For heavy metal derivatization, crystals were formed by seeding in drops of 75 nl of protein solution (18 mg/ml) and 75 nl of precipitant consisting of 0.2 Na Formate, 0.1M Bis-Tris-Propane, pH 7.5, 20% PEG 3350 and 10% Ethylene Glycol. The crystals were soaked in solution consisting of the mother liquor, supplemented with 1 mM Thiomersal for 60 min. In all cases, the crystals were cryo-protected using the well solution supplemented with 15% ethylene glycol and flash-frozen in liquid nitrogen.

### Data collection and structure determination

Native 2.6 Å dataset—diffraction data were collected from a single crystal at the Diamond synchrotron beamline I02 at a wavelength of 0.9795

Derivatized dataset—diffraction data were collected from a single crystal at the Diamond synchrotron beamline I03 at a wavelength of 0.9763

The protein crystallized in space group P1 21 1, with six monomers in the asymmetric unit.

Repeated attempts to solve the 2.6 A dataset by molecular replacement failed, presumably due to low homology between LACTB2 and the search models. A derivatized crystal dataset was collected and scaled to 3.2 Å. Eight mercury sites were unambiguously identified using SHELXD (49), with an anomalous signal extending to 6 Å. The structure was phased in a SAD experiment using SHARP (50). A substructure was built using PHENIX (51) AutoBuild. This substructure was manually rebuilt and then used to rigid body refine the 2.6 Å dataset in REFMAC (52). Several rounds of refinement and manual rebuilding was performed using BUSTER-TNT and Coot (53). The deposited structure was refined to a final resolution of 2.6 Å, R = 0.179, Rfree = 0.224 and assigned the PDB ID: 4AD9. The data collection and refinement statistics are presented in Table 1; a full validation report can be found in the supplementary materials.

**Table 1.** Data collection and refinement statistics

4AD9	
**Data collection**	
Space group	P 1 21 1
Cell dimensions	
*a, b, c* (Å)	107.47 95.69 135.76
α, β, γ (°)	90.0 112.84 90.0
Resolution (Å)	46.85–2.60 (2.67–2.70)*
*R*_merge_	0.07
*I*/σ*I*	2.12 (at 2.61)
Completeness (%)	99.2 (72.23–2.16)
Redundancy	10.8 (5.7)*
**Refinement**	
Resolution (Å)	2.60
No. reflections	77 582
*R*_work_/*R*_free_ (in high-resolution shell)	0.1775/0.2237 (0.2096/0.2653)*
No. atoms	14 209
Protein	13 126
Ligand/ion	28
Water	1055
*B*-factors	35.2
R.m.s. deviations	
Bond lengths (Å)	0.01
Bond angles (°)	1.14

*Values in parentheses are for highest-resolution shell.

### Synthetic RNA labeling

Labeling of the 5΄-end of the short synthetic RNAs (IDT) (Supplementary Table S2) was performed with T4 polynucleotide kinase (NEB) and [γ-^32^P] ATP, obtaining mono-phosphorylated substrates (54). The 3΄ end was labeled using T4 ligase and [^32^P]Cp, generating a labeled RNA containing an additional C at the 3΄ end (54). Full-length RNA molecules were purified by fractionation, using PAGE, followed by elution of the full length molecule from the gel by overnight incubation in RNA elution buffer (0.3 M NaOAc pH 5.5, 1.25 mM ethylenediaminetetraacetic acid (EDTA) and 0.1% sodium dodecyl sulphate) at 4°C.

### *In vitro* transcription

Body-labeled RNA was prepared using annealed DNA primers (IDT) (Supplementary Table S3). The first 20 nucleotides (nt) are attributed to the T7 promoter followed by the 40 nt template sequence. The complementary primers were heated for 2 min at 95°C and annealed by cooling at room temperature for 10 min. For body-labeled [^32^P]-RNA, the annealed dsDNA product was transcribed using T7 RNA polymerase and [α-^32^P]UTP (55). RNAs were resolved on 15% denaturing polyacrylamide gels, and the full-length products were eluted from the gel by overnight incubation in RNA elution buffer (described above) at 4°C.

### *In vitro* RNA degradation assays

*In vitro* RNA degradation assays were performed using the recombinant protein and either 5΄ [^32^P]-labeled, body-labeled, or 3΄ [^32^P]Cp-labeled RNA substrates. In a common reaction, a protein (7.5 μM) was incubated at 37°C with 0.08 μM RNA in a buffer containing 12.5 mM Tris–HCl pH = 7.4, 125 mM NaCl, 2.5% glycerol and 0.25 mM DTT for the times indicated in the figure legends. When relevant, 10 μM yeast tRNA (Sigma) was added to the reaction. Following incubation, the RNA was analyzed by denaturing PAGE and autoradiography.

### RNA binding assay

The ultraviolet (UV) light cross-linking of proteins to radio-labeled RNA was performed as previously described (56). Proteins were mixed with uniformly [^32^P]-labeled RNA and cross-linked at a UV cross-linker that was set at 1.8 joules (Hoefer), followed by RNA digestion with 10 μg RNase A and 30 units RNase T1, at 37°C, for 1 h. The proteins were then fractionated by sodium dodecyl sulphate-polyacrylamide gel electrophoresis (SDS-PAGE) and analyzed by autoradiography.

### GU-oligonucleotide cleavage and PNPase assay

For the (GU)_12_ oligonucleotide degradation assay, the substrate was 5΄ end-labeled using [γ-^32^P]ATP, as described above. Then, 1 μg yeast tRNA (Sigma) and either 0.001 U RNase A (sigma) and 0.1 U RNase T1 (sigma) were incubated with the radiolabeled (GU)_12_ RNA on ice for 3 min. Following incubation, the RNA was analyzed by denaturing PAGE and autoradiography. For the PNPase assay, incubation with nuclease P1 was for 30 min at 50°C in a buffer containing: 1 u/μl Nuclease P1, 0.2 mg/ml yeast tRNA, 20 mM sodium citrate (pH 9.0), 6.5 M urea and 1 mM ZnCl_2_. Incubation with RNase A (0.1 μg/ml) was on ice for 1 min with the addition of 0.4 mg/ml of yeast tRNA. Incubation with PNPase (15 μg/ml) was at 37°C for 30 min in buffer E containing 5 mM Pi (55). Following the first incubation, reactions were phenol extracted and EtOH precipitated before the second incubation with PNPase.

### Cell culture, transfection and RNA interference

HEK-293E cells were grown as a monolayer at 37°C, 5% CO_2_ in dulbecco's modified eagle's medium medium (Sigma) supplemented with 10% fetal calf serum, 2 mM L-glutamine, and 1% penicillin- streptomycin. A 27-mer LACTB2-specific siRNA duplex (IDT) (Supplementary Table S4) or 25-mer scrambled universal siRNA duplex (negative control) (IDT) was used for the transfection. Cells (1 × 10^6^) were transfected with 0.1 μM siRNA duplex on 60 mm plates using jetPRIME reagent (Polyplus) and harvested 48 h post transfection.

### RNA extraction, qRT-PCR and northern analysis

Total RNA was purified using Tri-Reagent (Sigma). The quantification of the RNA were performed using a NanoDrop spectrophotometer and gel electrophoresis. For qRT-PCR analysis, 2 μg total RNA were treated with DNase I (Invitrogen) and then reverse transcribed using random priming (Applied Biosystems). Then, 10 ng cDNA, 0.1 μM real-time primer set and SYBR green mix (Quanta Biosciences) were used in the qRT-PCR reaction for gene expression analysis (Bio-Rad CFX96). RNA blot analysis was performed, as described (57). Briefly, 5 μg total RNA was fractionated on a denaturing formaldehyde 1.2% agarose gel and blotted on to a nylon membrane. The RNA was UV cross-linked to the membrane and hybridized to [^32^P]-labeled oligonucleotides that complemented human mitochondrial transcripts (Supplementary Table S6). Hybridization was carried out at 57°C for 16 h followed by several washes and autoradiography.

### Mitochondrial isolation, fractionation and alkali treatment

Mitochondria were purified as previously described (58). Briefly, 1–3 ×10^9^ cells were harvested and homogenized in swelling buffer (10 mM Tris–HCl, pH 7.5, 10 mM NaCl and 1.5 mM MgCl_2_). The sucrose concentration was adjusted to 250 mM, and the nucleus fraction was removed by low-speed centrifugation. The mitochondria were washed twice to remove possible nuclear contaminants and were then pelleted at 15 000 × *g* for 15 min. The washing step was repeated to yield the final pellet of the mitochondria fraction. Fractions of the intermembrane space and the matrix of the mitochondria were analyzed by the proteinase K accessibly test as described (15). The protein profile (20 μg) was analyzed by immunoblotting using specific antibodies described below. Mitochondrial matrix extract was prepared from fresh bovine liver as described in (59). Alkaline sodium carbonate extraction was performed as described in (15). The mitochondrial fraction (100 μg proteins) was incubated in 100 mM Na_2_CO_3_ (pH 11.5) on ice for 30 min prior to a 30 min centrifugation at 100 000 × *g*, to yield the pellet fraction (intrinsic membrane proteins) and soluble fraction (peripheral proteins).

### Antibodies

Proteins were analyzed on a 12% SDS-PAGE, blotted to a nitrocellulose membrane and decorated with appropriate antibodies that were detected by chemo-luminescence using the ImageQuant (LAS4000) system. The following antibodies were used: LACTB2 (Sigma HPA044391 or Thermo Scientific PA5–32043), CPSF3L (Sigma HPA029025), GAPDH (Santa Cruz Biotechnology FL-335), Tom 20 (Thermo Scientific PA5 39247), HSP60 (Thermo Scientific PA5–12484), PNPase (Thermo Scientific PA5–22396), CYT C (Santa Cruz Biotechnology H-104) and subunit V of the ATP-synthase (60).

## RESULTS

### LACTB2 is a soluble monomeric protein that is located in the mitochondria matrix

**Figure 1.**
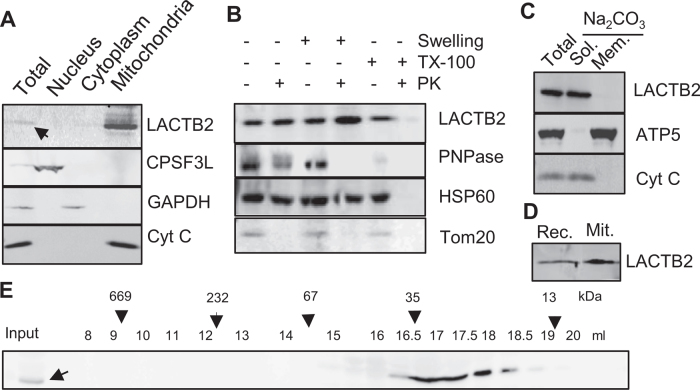
LACTB2 is a mitochondrial, soluble and monomeric protein. (**A**) Mitochondria localization. HeLa cells were disrupted and the nuclear, cytoplasmic and mitochondria-containing fractions were separated and analyzed for the localization of LACTB2, using an immunoblotting assay. The nucleus-located protein, CPSF3L, was used as marker for nuclear proteins. Glyceraldehyde-3-phosphate dehydrogenase (GAPDH) served as a cytosolic marker and cytochrome C (Cyt C) as a mitochondrial marker. (**B**) Immunoblot analysis of mitochondrial extract following Proteinase K accessibility assay. TX-100, Triton X-100. PK, Proteinase K. PNPase is a mitochondrial protein located mainly in the intermembrane space and a small amount in the matrix. HSP60 is a mitochondrial matrix protein. Tom20 is a mitochondrial outer membrane protein. (**C**) LACTB2 is a soluble protein. Immunoblotting analysis of mitochondrial extract following alkaline sodium carbonate (Na_2_CO_3_) extraction of soluble and peripheral membrane proteins. T, total extract. S, supernatant. P, pellet. Subunit 5 of the ATP synthase (ATP5) was used as a marker for intrinsic membrane proteins and Cyt C as a marker of soluble proteins. (D) Immunoblot analysis of the recombinant LACTB2 (Rec.) and the native protein in isolated mitochondria (Mit.) showing the same size on SDS-PAGE. Therefore, the mitochondria targeting signal is not cleaved upon entering the mitochondria and remain an integral part of the mature protein. (**E**) LACTB2 is present as a monomer. Soluble proteins of bovine mitochondrial matrix were extracted from bovine liver as described in the methods section. This extract was fractionated on a size exclusion column superdex 200 and analyzed for LACTB2, using an immunoblotting assay. The following proteins were used as size markers: Tyroglobulin (669 kDa), polynucleotide phosphorylase (PNPase) (232 kDa), BSA (67 kDa), β-lactoglobulin (35 kDa) and ribonuclease A (13 kDa).

MBL proteins displaying ribonuclease activity have been found in bacteria, archaea, chloroplasts and animal cells. In this work, we looked for such a protein in human mitochondria. The first described mitochondrial protein of this family, ELAC2, is responsible for processing of the 3΄ end of the mitochondrial tRNAs (17,61). However, ELAC2 does not seem to be involved in the processing or degradation of other RNA types in this organelle (62). Therefore, we asked whether there is an additional ribonuclease in the MBL protein family in human mitochondria. The study was initiated by searching the Mitoproteome database (44,45) to identify putative mitochondrial MBL proteins with predicted ribonuclease activity. The search revealed LACTB2, a nuclear-encoded protein with an unknown function, that is translated in the cytoplasm and predicted to be imported into the mitochondria. A literature search confirmed that LACTB2 is found in the mouse mitochondrial proteome (63,64) and in the human proteome project of mitochondria protein contents (65). An analysis using the mitochondria targeting prediction tools, MitoProt II and Target P (66,67), disclosed a 90% probability that LACTB2 harbors a mitochondrial localization signal of 27 amino acids at the N-terminus. To experimentally determine the localization of endogenous human LACTB2, HeLa cells were biochemically fractionated to nuclear, cytosolic and mitochondrial subcellular fractions, and the proteins were analyzed by an immunoblotting assay with specific antibodies. Figure 1A demonstrates that LACTB2 co-localized with cytochrome c (cyt c) to the mitochondria. LACTB2 was protected from degradation when the mitochondria were exposed to Proteinase K, even when mitochondria were swollen to rupture their outer membrane, indicating a localization in the matrix, where the majority of the mitochondrial transcripts are present and where RNA metabolism takes place (Figure 1B). The separation of the membrane and soluble proteins of the mitochondria using alkaline sodium carbonate treatment revealed that endogenous LACTB2 is a soluble protein that fractionates similar to cyt c (Figure 1C). As described above, a putative mitochondrial localization signal of 27 amino acids has been identified. Nevertheless, we could not produce the recombinant protein without the N-terminal 27 residues in a soluble form, and the analysis of the structure of the full length protein revealed these residues to be an integral part of the mature protein (see below). Indeed, the full length recombinant and the mature proteins co-migrated in SDS-PAGE, indicating that the 27 amino acids of the N-terminus remain integral part of the mature protein (Figure 1D).

Given its localization in the mitochondria, we tested whether LACTB2 forms complexes with other proteins within the mitochondria. Figure 1E presents the size exclusion chromatographic profile of soluble proteins of the mitochondrial matrix purified from bovine liver. An immunoblotting analysis using specific antibodies found that LACTB2 eluted at its monomeric molecular weight of 33 kDa, which is similar to the behavior of recombinant LACTB2 purified from *E. coli*. Together, these results confirm that LACTB2 is localized to the mitochondria matrix as a soluble monomeric protein.

### LACTB2 is a member of the MBL superfamily

**Figure 2.**
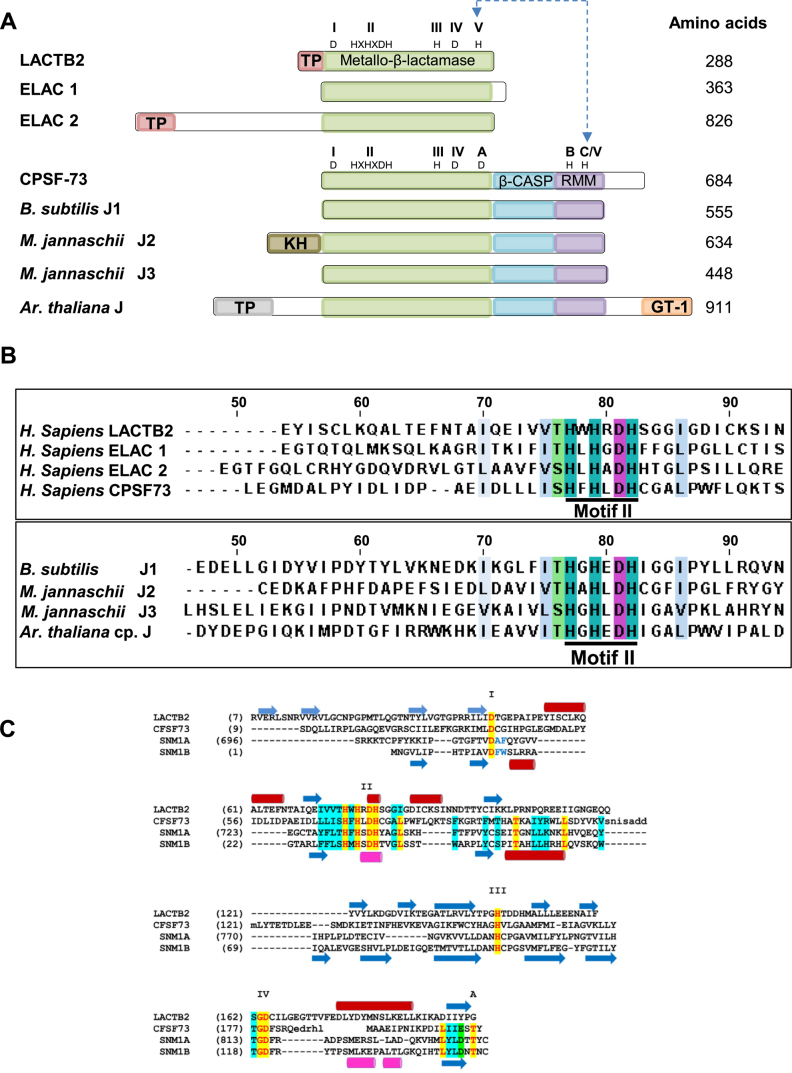
LACTB2 is a member of the metallo-β-lactamase (MBL) superfamily. (**A**) Domain analysis comparison of LACTB2, ELAC1, ELAC2, CPSF-73 of human, as well as bacterial, archaeal and plant proteins that are members of β-CASP MBL subfamilies. The core domains of the following proteins were aligned: human metallo-β-lactamase protein like 2 (LACTB2) (Q53H82), human cleavage and polyadenylation specificity factor 73 (CPSF-73) (Q9UKF6), human nuclear tRNAseZ (ELAC1), human mitochondrial RNaseZ (ELAC2) (Q9BQ52), bacteria *Bacillus Subtilis* RNase J1 (*B. subtilis* J1) (Q45493), archaea *Methanocaldoccous jannaschii* RNase J2 and J3 (*M. jannaschii* J2 and *M. jannaschii* J3) (MJ1236, MJ0861), and plant *Arabidopsis thaliana* RNase J that is located in the chloroplast (*A. Thaliana* J) (Q84W56). The conserved motifs of the MBL, β-CASP and RRM (I–IV; A–C) are indicated in green, blue and purple, respectively, along with the diagnostic amino acid residues. The MBL and β-CASP proteins were mapped based on their structural alignment (29,32). Motif V of LACTB2, ELAC1 and ELAC2 shares a linear sequence alignment with Motif C of the β-CASP proteins, which is represented as C/V. Mitochondria and chloroplast transit peptides are colored in pink and gray, respectively. The archaeal N- terminal region corresponds to the KH RNA-binding domain (85) and the plant C-terminal region includes a putative GT1 DNA-binding domain (86). The number of amino acids of each protein is indicated to the right. (**B**) Alignment of the amino acid sequence of Motif II of LACTB2 and several MBL ribonucleases. CLUSTAL W and JalView were used for the alignment. The numbers above the amino acid sequence indicate the position of amino acids in human LACTB2. Signature MBL motif II, HXHXDH, is underlined with a thick black line and His (H) residues are highlighted in cyan and Asp (D) residues in purple. The intense cyan and purple colors represent the highest degree of conservation among the eight proteins while pale blue and green colors signify a lower degree of conservation. Uncolored residues represent no conservation within the annotated threshold. (**C**) Structure-based alignment of the MBL domains of LACTB2, CPSF73 and the DNA exonucleases SNM1A and SNM1B (all from humans). Secondary structural elements (blue arrows: β strands; red cylinders: α-helices) are shown above the alignment for LACT2B and below for CPSF73. The Roman numerals I–IV indicate the conserved MBL motifs, and the specific residues are indicated highlighted in yellow; less conserved sequences are highlighted in cyan. The alignment ends where the structures diverge (β-CASP region in CPSF73, SNM1A and SNM1B, and the unrelated C-terminal region of LACTB2).

While no annotations have been assigned regarding its activity and biological function, we hypothesized that human LACTB2 performs a ribonucleolytic activity similar to several other MBL proteins. Figure 2A displays the core domain alignment of LACTB2 with MBL ribonucleases from the three domains of life. Specifically, the LACTB2 MBL core domain, including motifs I-IV, resembles the mitochondrial and nuclear tRNA 3΄ end processing endoribonucleases of the ELAC subfamily, RNase Z. The conserved motifs are well observed for β-CASP ribonucleases, such as the human CPSF-73 (40) and RNase J from archaea, bacteria and the plant chloroplast (37,39,42,68). However, the CASP (motifs A-C) and RMM (RNA-metabolizing MBL) domains are restricted to the β-CASP subfamily and are not found in LACTB2 or the ELAC subfamily. As expected, multiple sequence alignment conservation, focusing on the signature motif II- HXHXDH (Figure 2B), was observed throughout the above aligned proteins. An alignment of the MBL domains of LACTB2 with three other human MBLs (CPSF-73 and the DNA exonucleases SNM1A and SNM1B; Figure 2C) shows the location of the other motifs.

To better understand the function and enzymatic mechanisms of LACTB2, we solved a crystal structure of the recombinant protein. The full-length protein, including the putative mitochondrial targeting sequence, was expressed in *E. coli*. LACTB2 crystallized in space group P 1 21 1 and diffracted to 2.6 Å. A substructure was solved using single isomorphous replacement with anomalous scattering (SIRAS), and was then used to solve the native dataset by molecular replacement. There are six molecules of LACTB2 in the asymmetric unit, arranged as two trimers. Although PISA analysis (69) suggest that the trimer may be a biological unit, the protein purified as a monomer and showed no evidence of oligomerization in solution (Figure 1E). At this point, it is not clear if the trimeric structure is an artifact of crystallization.

**Figure 3.**
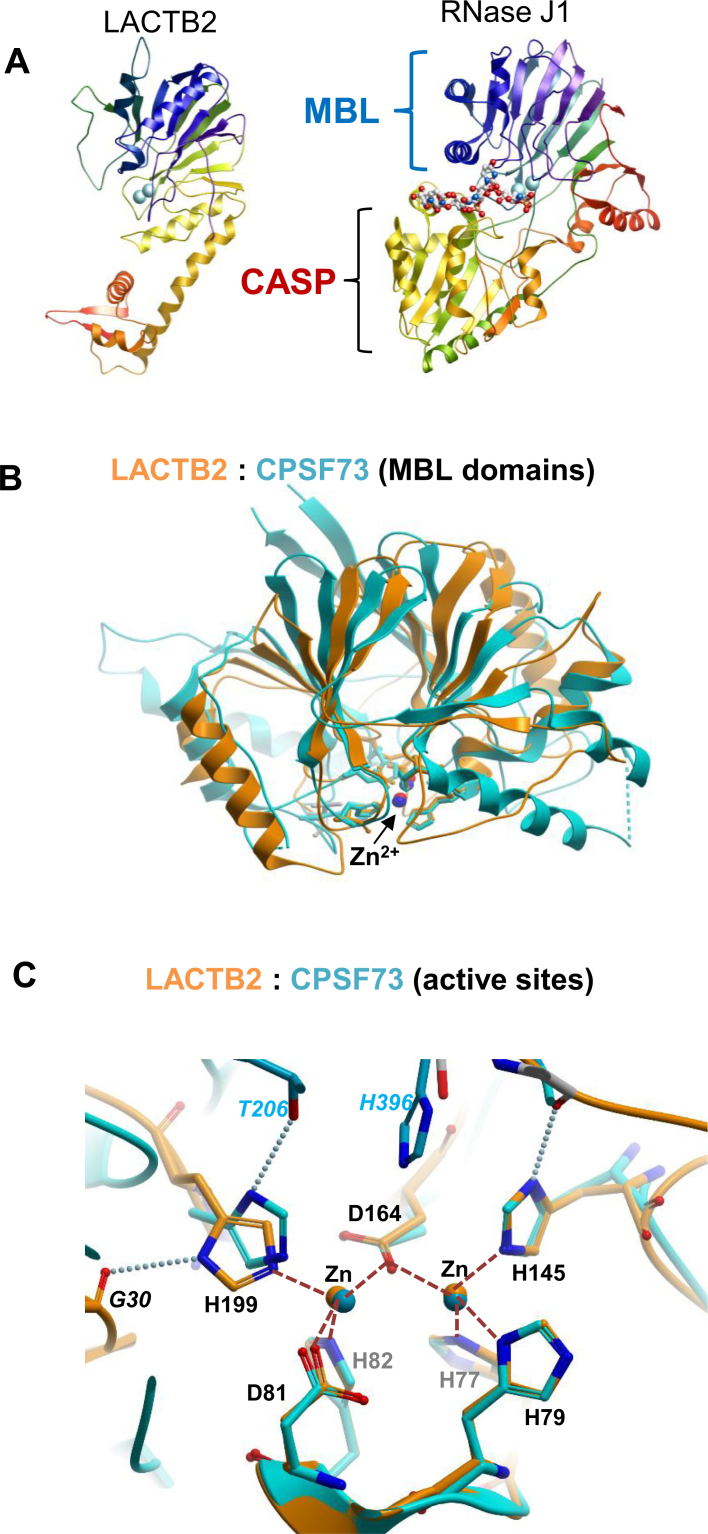
Crystal structure of LACTB2 and comparisons with β-CASP ribonucleases. (**A**) Comparison of the overall fold of LACTB2 and RNase J1 (PDB ID: 3T3N). The chains are colored in blue to red from the N- to the C-terminal and the Zn ions are shown as cyan spheres. The RNase J structure also includes a bound RNA oligonucleotide, shown in stick representation. The common MBL fold is shown on the top in both proteins, while the CASP domain is present only on RNaseJ. The chain is colored as a rainbow spectrum from the N (blue) to C (red) terminus. (**B**) Superposition of the MBL domains of LACTB2 (aa 1–211 orange) and CPSF-73 (PDB ID: 2I7T, residues 9–207 and 395–459; cyan). (**C**) Closeup of the active sites of LACTB2 (orange) and CPSF-73 (cyan). The numbers of the homologous residues from LACTB2 (shown in the figure) and CPSF73 are, respectively, Motif II: His 77/71, His 79/73, Asp 81/75 and His 82/76. Motif III: His 145/158. Motif IV: Asp 164/179, and motif C/V: His 199/418. Motif I (Asp 46/39), which helps to orient His 82/76 but does not coordinate the zinc ions, is not shown. Motif A (Thr 230 in CPSF73), which helps to orient His 418, is conserved in SNM1A/B, but absent in LACTB2; His 199 of LACTB2 is instead supported by an interaction with the main-chain carbonyl of Gly 30. The Zn^2+^ ions are presented as spheres; coordination bonds as dashed orange lines, and selected hydrogen bonds are depicted as dotted lines. The His 396 residue belonging to motif B of the β-CASP domain of CPSF-73 that is lacking in LACTB2, is also indicated.

The N-terminal (200 residues) of LACTB2 is a typical MBL fold, with a central double β sheet sandwich flanked by helices and loops (Figure 3A). The putative mitochondrial targeting sequence (aa 1–27) is an integral part of this fold, providing the first two strands of the β-sheet (this probably accounts for the insolubility of a recombinant protein lacking aa 1-27). Compared to ribonucleases such as CPSF73 and RNase J, LACTB2 lacks the CASP domain, and instead has a smaller C-terminal extension. β-CASP proteins contain a deep groove around the di-Zn active site, flanked by the MBL and CASP domains. In contrast, LACTB2 has a shallow surface, which may have functional consequences such as promiscuity of substrates and lack of processivity (Figure 3A and Supplementary Figure S1C). The unique C-terminal domain of LACTB2 is reminiscent of a sequence insert within the MBL domain of arm of tRNase Z, which plays a role in recognition of the complex structure of the pre-tRNA substrate (the exosite; (70)). While the C-terminus of LACTB2 does not have obvious sequence or structural similarity with the exosite of tRNase Z, it remains to be seen if LACTB2 relies on the C-terminal domain for activity or recognition.

To further investigate the conservation of functional elements between LACTB2 and other MBL RNases, we superposed the MBL domain of LACTB2 (aa 1–211) with the MBL domain of CPSF-73 (PDB ID: 2I7T, residues 9–207 and 395–459) (40) (Figure 3B). The structures align with RMDS = 2.7 Å, with a similar topology of the core β-sandwich and more variance in the flanking helices. Note that the two Zn^2+^ ions, denoted as red (LACTB2) or blue (CPSF-73) spheres at the bottom of the structure, are at nearly identical positions (within 0.3 Å). A closeup of the catalytic regions (Figure 3C) shows a virtually identical spatial arrangement of the conserved Zn^2+^—coordinating residues H77/H71, H79/H73, D81/D75, H82/H76, H145/H158 and D164/D179, of LACTB2/CPSF-73, respectively (RMSD for the Zn^2+^-coordinating residues is 0.7 Å). An additional Zn^2+^ coordinating residue in β-CASP region of CPSF-73, H418, defined as motif C/V, is substituted by a non-homologous his residue (H199) in LACTB2. The presence of a histidine residue at the C/V motif is thought to be a distinguishing feature of MBL ribonucleases, and is absent in MBL deoxyribonucleases such as SNM1A and SNM1B (71). Comparisons with other MBL ribonucleases RNase J and tRNase Z (Supplementary Figure S1) reveal a similar conservation of the spatial arrangement of the active sites. Together, these results support the hypothesis that LACTB2 is a ribonuclease and prompted us to experimentally examine this possibility.

### Recombinant LACTB2 displays endoribonucleolytic activity

**Figure 4.**
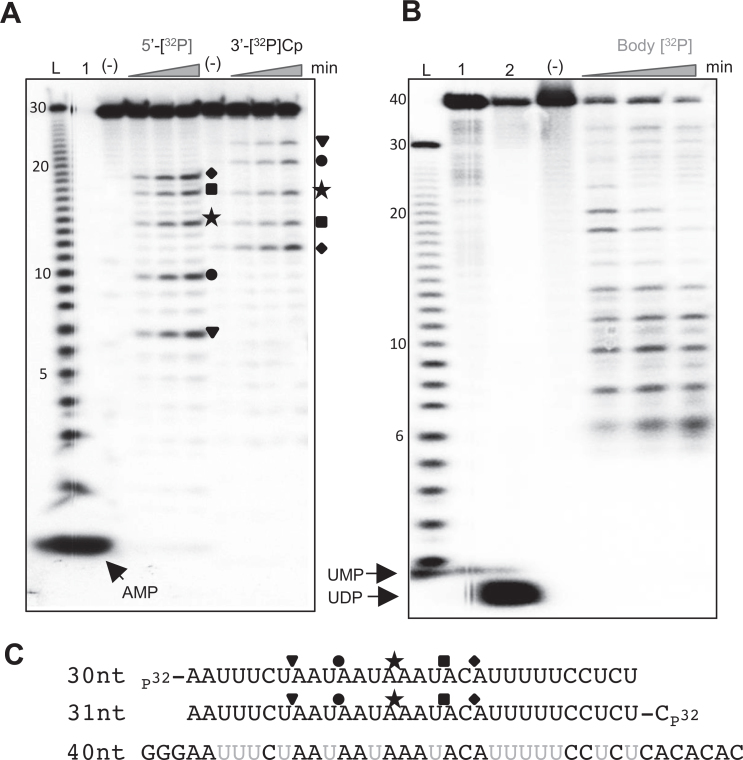
Recombinant LACTB2 displays endoribonuclease activity. (**A**) LACTB2 is an endoribonuclease. Recombinant and purified LACTB2 was incubated with 5΄ end [^32^P], or 3΄ end [^32^P]Cp, radiolabeled 30 nt-long RNA and unlabeled yeast tRNA. The reaction conditions included incubation at 37°C for 15, 30 and 60 min, followed by the addition of formamide dye and analysis by denaturing gel and autoradiography. Lane (L): nucleotide ladder created by alkaline hydrolysis of the substrate RNA. Lane (1): [^32^P]—AMP marker of 5΄ [^32^P]-labeled RNA, created by digestion with RNase One. Lane (−): RNA incubation for 60 min, with no addition of protein. Forms of the same shape presented to the right of the autoradiogram indicate the matching cleavage products of the complementary 5΄ and 3΄ RNAs and can be identified at the sequences presented below the figure, in panel **C**. (**B**) LACTB2 does not display exoribonucleolytic activity. LACTB2 was incubated with 40 nt body-labeled [^32^P]-UTP RNA substrate, with the nucleotide sequence shown in panel C. Lane (L): nucleotide ladder described in panel A. Lane (1): [^32^P]-UMP marker, prepared by the digestion of body-labeled RNA, using *Methanocaldoccous jannaschii* RNase J3, at 60°C for 1 h (42). Lane (2): [^32^P]-UDP marker, obtained by digesting body-labeled RNA with *Escherichia coli* PNPase. Other lanes are labeled as described for panel A. (C) RNA sequence of the substrates. The cleavage sites corresponding to the shape shown in panel A are indicated. The uridines labeled in the body-labeled RNA used in the experiment described in panel B are colored gray.

To assess whether LACTB2 displays ribonuclease activity, recombinant LACTB2, expressed in bacteria and purified to homogeneity, was used in RNA degradation assays. The recombinant LACTB2 was incubated with 30-nt-long, AU-rich, [^32^P]-RNA as a substrate, and degradation products were analyzed by denaturing gel electrophoresis and autoradiography. Several degradation products accumulated during the incubation period, indicating that LACTB2 is a ribonuclease (Figure 4A). To define whether the protein harbors endo- and/or exoribonuclease activities and to determine the direction of degradation, the RNA substrate was radioactively labeled at either its 5΄ or 3΄ end. Figure 4A displays five major 7, 10, 14, 17 and 19 nt-long cleavage products (see the sequence in Figure 4C), when the RNA was labeled at the 5΄ end. Accordingly, their complemented 24, 21, 17, 14 and 12 nt-long cleavage products were detected when the RNA was labeled at the 3΄ end (note that 3΄ end labeling involved the addition of a cytosine nucleotide to the RNA substrate). Because the cleavage patterns of the 5΄ and 3΄ end-labeled RNA complemented each other and no mononucleotides accumulated, we concluded that LACTB2 is an endoribonuclease. In addition, there does not seem to be a progressive decrease in average fragment size, indicating a predominantly single-hit cleavage reaction with little, if any, processivity. Other proteins of the MBL family, such as the bacterial and chloroplast RNase J, exhibit dual endo- and 5΄-3΄ exonucleolytic activity (39,72). There is a possibility that the 3΄ phosphate added when labeling RNA with pCp and T4 RNA ligase may inhibit a 3΄-5΄ exonuclease activity, as seen with PNPase. To test whether LACTB2 is exclusively an endonuclease or a dual endo/exonuclease, we incubated the protein with 40-nt RNA which was internally-labeled with [^32^P]UTP (Figure 4B). The pattern of cleavage was very similar to that obtained with end-labeled RNA (Figure 4A), and no mononucleotide accumulation was observed, confirming that LACTB2 is not an exoribonuclease. Together, these results demonstrate that, as predicted from the structural similarity to the active site of human CPSF73, human LACTB2 is a endoribonuclease.

**Figure 5.**
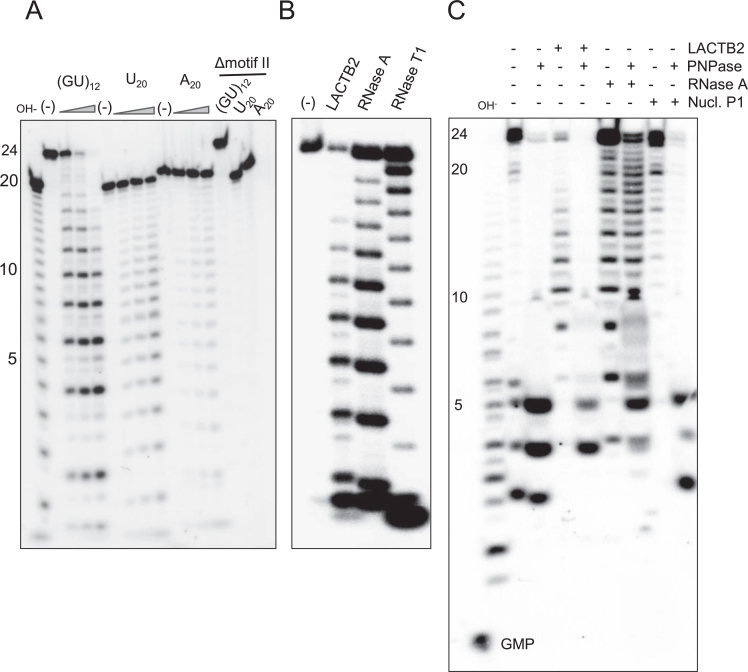
Recombinant LACTB2 prefers U, but not G or poly(A). (**A**) LACTB2 was incubated for 15, 30 and 60 min, at 37°C with poly(GU)_12_, poly(U)_20_ or poly(A)_20_, in the presence of yeast tRNA. The RNA substrates were labeled with [^32^P] at the 5΄ end. Lane (L): Nucleotide ladder prepared by alkaline hydrolysis of poly(U)_20_. Lane (−): RNA substrate incubated with no protein for 60 min. Lanes under the triangle—incubation for 15, 30 and 60 min. Lanes labeled ΔmotifII: the RNAs were incubated for 60 min with the Δmotif II mutated protein in which the six amino acids of motif II were deleted (see Figure 7). Following incubation, the RNA was purified and analyzed by denaturing PAGE and autoradiography. (**B**) Poly(GU)_12_ was digested in the presence of yeast tRNA with LACTB2, RNase A (cleaves following U) and RNase T1 (cleaves following G). Lane (−) is the same as in panel A. (**C**) LACTB2 cleavage products have 3΄-OH termini and therefore are sensitive to digestion by the exoribonuclease PNPase. 5΄ [^32^P] (GU)_12_ RNA was digested with the enzymes as indicated on the top. When using two enzymes, the RNA was purified by phenol extraction and EtOH precipitation between the incubations. The full length 24 nt RNA contains a 3΄-OH and therefore is sensitive to PNPase. The cleavage products of RNase A have 3΄-phosphate while those of Nuclease P1 contain 3΄-OH. Some leakage of the digestion products of PNPase to the lane of no protein (−) happened when the gel was loaded with the samples. Nucl. P1: Nuclease P1.

To determine the nucleotide preference of the ribonucleolytic activity of LACTB2, the protein was incubated with either 5΄ end-labeled poly(U)_20_, poly(A)_20_ or poly(GU)_12_ RNA substrates. While very low endoribonucleolytic activity was observed with poly(U) and even less with poly(A), a distinct cleavage pattern was observed with poly(GU)_12_ substrates (Figure 5A). Because the incubation of LACTB2 with poly(GU)_12_ RNA resulted in cleavage at every other nucleotide, we determined whether this cleavage followed the nucleotide G or U. To this end, the RNA was digested by RNase A, cleaving after pyrimidine residues (U or C) or by RNase T1, that cleaves following G. Figure 5B shows that the LACTB2 cleavage pattern was identical to that of RNase A, indicating a preferred cleavage after a U residue. Similarly, when analyzing a poly(AC)_12_ RNA substrate, the cleavage pattern of LACTB2 is similar to that of RNase A, cleaving this substrate following C (Supplementary Figure S2). Combining with the cleavage points observed in Figure 4A, we conclude that LACTB2 has a preference for cleavage 3΄ to purine-pyrimidine dinucleotide sequences.

MBL ribonucleases, containing a Zn^2+^ at the active site, typically leave a 5΄-phosphate and 3΄-OH on the cleavage products. To verify that the cleavage products of LACTB2 are characterized with 3΄-OH, we used the PNPase assay. PNPase is a 3΄ to 5΄ processive exoribonuclease that digest 3΄-OH, but not 3΄-phosphate RNAs (73,74). Therefore, the LACTB2 cleavage products, having 3΄-OH, should be sensitive to the degradation activity of this enzyme. The results of such an experiment, presented in Figure 5C, disclosed that the LACTB2 cleavage products were degraded by PNPase, similar to those of the enzyme Nuclease P1. However, the cleavage products of RNase A, that are characterized with 3΄-phosphate, were resistant to digestion by PNPase (Figure 5C). As described before for PNPase, this enzyme does not digested the RNA molecule to the final nucleotide, leaving oligoribonucleotides of 3–5 nt in length (75).

**Figure 6.**
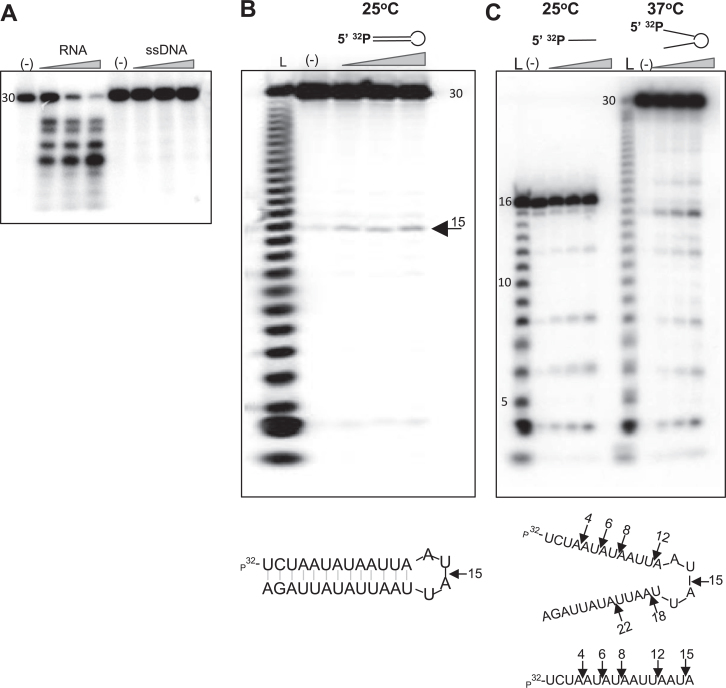
Recombinant LACTB2 cleaves ssRNA but not dsRNA or ssDNA. (**A**) LACTB2 does not cleave ssDNA. LACTB2 was incubated with 5΄ [^32^P] 30 nt-long RNA or ssDNA of the same sequence for 15, 30 and 60 min at 37°C. Lane (−): RNA or ssDNA incubated for 60 min, with no added protein. Lanes under the triangle: incubation for 15, 30 and 60 min. (**B**) A stem-loop structured RNA is cleaved by LACTB2 at the loop. LACTB2 was incubated with 5΄ [^32^P] 30 nt stem-loop-shaped RNA, as presented below the figure. The reaction was incubated at 25°C, a temperature in which the stem is formed, for 15, 30 and 60 min in the presence of yeast tRNA. Lane (L): nucleotide ladder of the 30 nt RNA. Lane (−): incubation for 60 min, with no added protein. Lanes under the triangle: incubation for 15, 30 and 60 min. The sequence and predicted stem-loop structure formed at 25°C is shown at the bottom. (**C**) LACTB2 cleaves the substrate at a temperature in which the stem-loop structure is not formed. LACTB2 was incubated at 25°C in the presence of yeast tRNA with a linear 16 nt RNA corresponding to the nucleotide sequence of the stem of the molecule used in (B), in order to show the presence of cleavage sites in this sequence. In addition, the same reaction presented in panel B, was repeated at 37°C, where the stem-loop structure is not predicted to be formed. The sequence and structure of the RNA at 37°C are shown below the autoradiogram. Black arrows indicate the cleavage sites.

**Figure 7.**
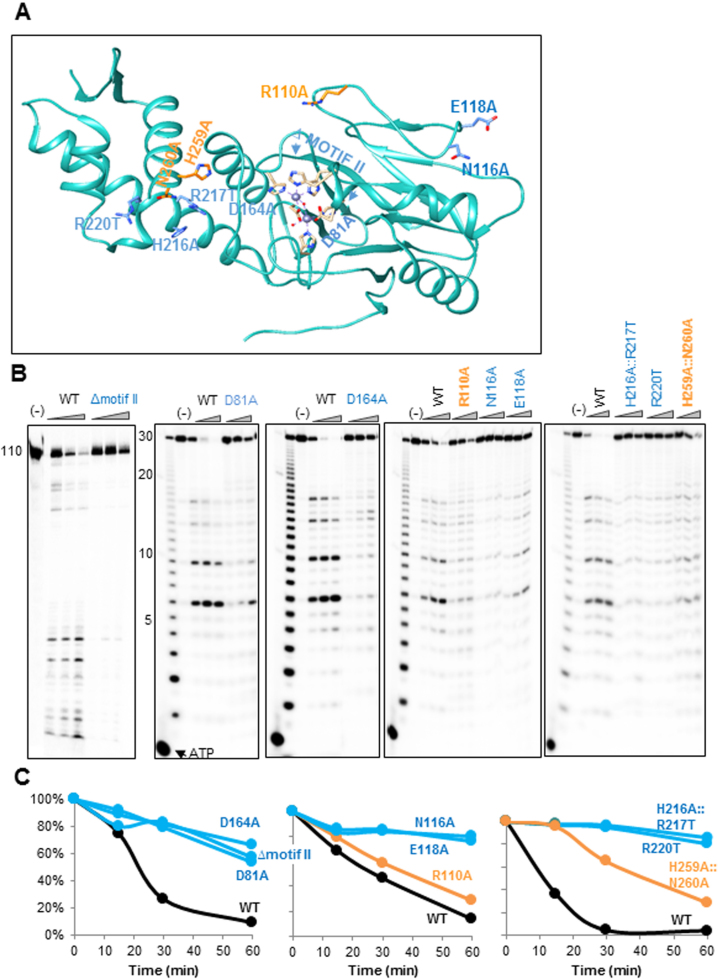
Mutation of certain amino acids in the vicinity of the catalytic site, as well as at other locations, significantly inhibits the ribonucleolytic activity. (**A**) The locations of nine LACTB2 mutations introduced in this work are indicated in the structural image of the protein, created using Chimera. The mutated amino acids that significantly inhibited the endoribonucleolytic cleavage activity are colored in blue, and those not impairing the activity are colored in orange. The ΔMotif II mutation is a deletion of the entire motif II (amino acids HWHRDH). The other mutations, located in the vicinity of the cleavage catalytic site, were D81A and D164A. Additional mutated amino acids were: R110A, N116A, E118A, H216A::R217T, R220T and the double mutant H259A::N260A. The two zinc ions are presented as purple spheres and hydrogen bonds are presented as with purple dashed lines. (**B**) Non-mutated (WT) and LACTB2 mutants ΔMotif II, D81A, D164A, R110A, N116A, E118A, H216A::R217T, R220T, H259A::N260A were incubated with 5΄ [^32^P] −110 nt RNA corresponding to the mitochondrial COX1 transcript (left panel) or 30 nt RNA described in Figure 4 at 37°C, followed by the addition of formamide dye and analysis by denaturing gel and autoradiography. Deca RNA Marker was fractionated in the first lane to the right and the nucleotide ladder of the 30 nt RNA substrate is shown in the next lane. Lane (−): RNA incubation for 60 min with no added protein. Lanes under the triangle: incubation for 15, 30 and 60 min. (**C**) Kinetic graphs displaying the quantification of the disappearance of the full-length substrate RNA, when incubated with LACTB2 WT or the mutated proteins. The amount of RNA present in the lane marked (−) was taken as 100%. WT is colored in black, RNA cleavage in mutants displaying similar activity to WT is colored in orange. The activity of mutated proteins in which a significant inhibition was observed is colored in blue.

Additional evidence for the 3΄-OH termini of the cleavage products could be observed by the slight shift in the migration of the LACTB2 products as compared to those generated by RNase A. The latter run faster on the gel due to the additional phosphate group at the 3΄ end (Figure 5B). However, in our hands most of the gels did not reveal differences in the migration of the cleavage products (Supplementary Figure S2 and Figures 5–7). As a Zn^2+^ containing MBL ribonuclease, 50 mM EDTA and 20 mM *o*-phenanthroline, but not 50 mM imidazole, inhibited the RNA cleavage activity (Supplementary Figure S3).

### LACTB2 cleaves ssRNA but not dsRNA or ssDNA

The β-CASP family includes three highly conserved amino acids motifs: A, B and C corresponding to Glu/Asp, His and His/Val residues, respectively, that are within the vicinity of the MBL active site (Figure 2) (32). The conserved amino acid in Motif C is correlated with substrate preference, where His is for RNA nucleases, and Val is for DNA nucleases (32). In addition to their natural RNA-degrading activity, β-CASP ribonucleases, such as human CPSF73, *M. jannaschii* RNase J3, and *Pyrococcus abyssi* and *Thermococcus kodakaraensis* RNase J, have the ability to degrade ssDNA (41,42,68). LACTB2 is an MBL protein that lacks the β-CASP-RMM domain, yet, there is a His residue (His 199) at a location corresponding to motif C/V of the β-CASP proteins (Figures 2A and 3C). LACTB2 failed to cleave a ssDNA bearing a sequence identical to a ssRNA it successfully cleaved (Figure 6A); increasing the amount of the protein did not change this result (data not shown). Thus, we concluded that LACTB2 is restricted to RNA degradation. To explore whether LACTB2 can cleave double-stranded RNA, two RNA substrates were designed using RNA-fold (76): a 30-nt-long stem-loop RNA, and a 16-nt ssRNA with the same sequence as the first 16-nt of the stem. (Figure 6B bottom). Because the secondary structure of the dsRNA is formed at 25°C (calculated Tm is 32.8°C), we first assayed the cleavage activity at that temperature. Figure 6B shows a single distinct cleavage at nucleotide 15, which corresponded to the single-stranded loop structure of the dsRNA molecule. Two controls were performed before concluding that LACTB2 does not digest double-stranded RNA. First, confirming that LACTB2 is active at 25°C, and second, that the substrate RNA, while not in a double-stranded form, is cleaved at several sites at 37°C, a temperature where the stem structure is not predicted to be formed (Figure 6C). We interpreted these results as follows: at 25°C, the stem-loop structure is formed, and the stem structure is protected from cleavage by LACTB2. However, at 37°C, the stem is not formed, and the RNA is single-stranded and sensitive to the cleavage activity of LACTB2. Taken together, these findings indicate that LACTB2 cleaves ssRNA but not dsRNA or ssDNA.

### Mutations of certain amino acids reveal key residues involved in ribonucleolytic activity

Nine mutations were designed to replace amino acids that were predicted as being involved in ribonucleolytic activity (Figure 7A). Three mutations were introduced in the vicinity of the putative active site of the MBL domain. In the first mutation, the entire motif II, composed of the amino acids HWHRDH, was deleted (Δmotif II). In the second mutation, the aspartic acid of motif II was replaced with alanine (D81A). In the third mutation, located in the vicinity of the putative active site, the aspartate of motif IV was replaced with alanine (D164A). Six other mutations (R110A, N116A, E118A, H216A::R217T, R220T and H259A::N260A) replacing amino acids along a possible RNA-binding groove were introduced, and the corresponding proteins were examined for their ribonucleolytic activity. The activity of the mutated proteins was analyzed by incubating them with [^32^P]-labeled RNA (Figure 7B). Quantitation of the amount of remaining full-length RNA substrate was used as the relative degree of ribonucleolytic activity of the mutated protein compared to wild-type LACTB2 (Figure 7C).

The catalytic center of MBL enzymes is defined by the presence of two Zn^2+^ ions that are coordinated by five or six histidines, two aspartic acids and a water molecule (29). Accordingly, the activity of MBL ribonucleases was severely inhibited when the six amino acids of motif II, HWHRDH, were mutated (Figures 7 and 5), indicating the importance of these residues in the formation of the active site, as described for RNase J proteins and CPSF-73 (36,40,42,72). Similarly, three mutations that modified amino acids hypothesized to build the catalytic site of the ribonucleolytic activity were introduced. Asp81 is part of motif II and coordinates one of the two Zn^2+^ ions (Figure 7A). Changing this amino acid to alanine severely impaired the RNA degradation activity as is the case in *Bacillus* RNase J1 and human CPSF-73 (Figure 7B–C) (40,72). Mutating Asp164 (motif IV), which is responsible for bridging the two Zn^2+^ ions, to alanine, resulted in significant reduction in ribonucleolytic activity (Figure 7B and C). This result resembles that obtained when the corresponding amino acid was mutated in *A. thaliana* RNase Z protein (77). These results defined the predicted ribonucleolytic active site, which proved similar to those of other MBL ribonucleolytic proteins.

The next six mutations targeted several hydrophilic residues on the surface of the protein. While changing the positively charged mutation of R110 to Ala led to no change in the ribonucleolytic activity, the N116A, E118A, double mutant H216A::R217T and R220T severely inhibited the ribonucleolytic activity of LACTB2 (Figure 7B and C). The double mutant H259A::N260A displayed a degree of degradation activity that was between that of the fully active and the inactive forms of the protein.

Because the last described six mutations are not located in the vicinity of the predicted active site and four (N116A, E118A, double mutant H216A::R217T and R220T) severely inhibited the degradation activity, we hypothesized that these residues may function in either RNA binding or protein structure curvature. Unlike the well-described ribonucleolytic active site of MBL ribonucleases, it remains unknown if additional RNA-binding domains exist. Several of the archaeal CPSF proteins include a KH RNA binding motif recognizing specific nucleic acid sites (78–80). However, no specific RNA-binding consensus was found in the *Bacillus* RNase J1 or mammalian CPSF-73, in addition to the active site binding pocket (40,72,81). To explore the RNA-binding properties of the various LACTB2 mutants, we performed a UV-crosslinking assay using [^32^P]UTP body-labeled RNA (Supplementary Figure S4). The degree of UV-crosslinking signal indicates different manners of RNA binding to LACTB2 WT and mutated proteins. Δmotif II, displayed severely inhibited RNA binding activity, manifested by absence of a UV-crosslinking signal (Supplementary Figure S5A). Additionally, the protein with a mutated motif IV (D164A), which displayed a significant reduction in RNA cleavage activity, showed approximately half of the UV-crosslinking signal observed for the WT protein. These two observations are compatible with the deletion of motif II and mutation on motif IV of *A. thaliana* RNase Z when analyzing RNA binding and processing activity (77). Mutating D81A of motif II, which displayed impaired ribonucleolytic degradation activity, presented the same UV-crosslinking signal as that of WT, suggesting that this residue is not essential for RNA binding. Mutations R110A, N116A, E118A and H259A::N260A showed no change in the UV-crosslinking signal, suggesting similar binding affinity compared to WT, indicating that the corresponding amino acids are not essential for RNA binding, even though the ribonucleolytic activity of N116A and E118A was significantly reduced. H216A::R217T and R220T, demonstrated a very poor UV-crosslinking signal that may reflect compromised binding affinities to RNA. This observation is compatible with the poor ribonucleolytic activity observed for these two mutants. Examination of the electrostatic surface structure of LACTB2 (Supplementary Figure S4B) revealed a positive patch formed, in part, by these amino acids that may function in anchoring the negatively charged RNA to the protein, thereby acting as an additional RNA binding domain that is the first to bind the molecule and then direct it to the ribonucleolytic active site.

Taken together, these results suggest an extended RNA-binding site that plays an essential role in the RNA-degrading activity of LACTB2. Deletion of motif II (Δmotif II), the mutation on motif IV (D164A) and two mutations on the surface of the protein, H216A::R217T and R220T, lead to reduced RNA binding, resulting in a poor RNA degradation activity of the protein. The impaired RNase activity of the mutants N116A and E118A does not correlate with a reduction in RNA binding in the cross-linking assay, so the role of these residues is less clear. Further investigations and perhaps the crystallization of this protein with an RNA molecule are required to fully understand the nature of LACTB2 RNA binding. The involvement of residues outside the catalytic site in nuclease activity has been recently demonstrated in the MBL DNA repair nucleases SNM1A and SNM1B (71), indicating that an elongated RNA/DNA binding groove is a common feature of MBL nucleases.

### Decreased expression of LACTB2 result in significant cellular damage and morphological changes

**Figure 8.**
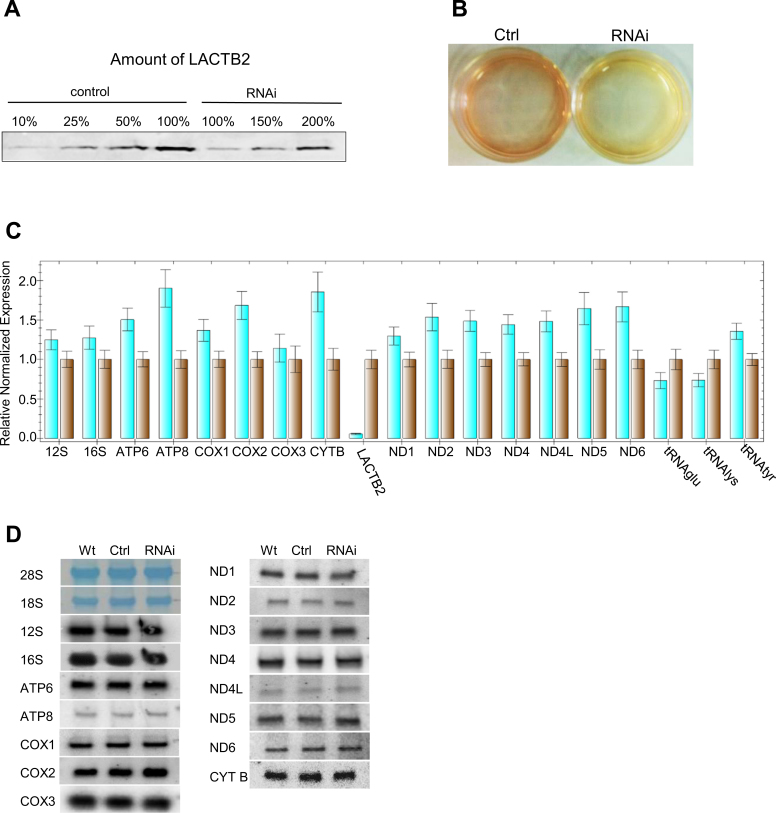
Downregulation of LACTB2 expression resulted in modest accumulation of mitochondrial transcripts. (**A**) Quantitation of the amount of LACTB2 in the siRNA treated cells. siRNA duplex targeted for LACTB2 or scrambled siRNAs duplexes, as a negative control, were transfected into HEK-293 cells. Cells were collected 48 h post-transfection and the amount of LACTB2 was determined using immunoblotting assay. Proteins from equal number of cells were loaded in the lanes labeled 100%. Proteins from 50, 25 and 10% of the control and 150 and 200% of the siRNA LACTB2 treated cells were analyzed for the amount of LACTB2, in order to determine the degree of down-expression. (**B**) Medium acidification due to treatment with siRNA duplex, 48 h post-transfection. Ctrl—cells treated with scrambled siRNAs duplexes as a negative control. RNAi—cells treated with siRNA duplex targeted to LACTB2. (**C**) The amount of mitochondrial transcripts in the cells treated with the siRNA duplex targeted to LACTB2 (turquoise bars), or with scrambled siRNAs duplexes as a negative control (brown bars), was analyzed by qRT-PCR. The GAPDH transcript was used as the reference gene for normalization of the expression. The error bars display the standard errors of at least three independent experiments. (**D**) Northern blot analysis of HEK-293 cells that were not transfected (WT), transfected with scrambled siRNAs as control (Ctrl) or transfected with LACTB2 siRNA (RNAi). Hybridizations were performed with probes specific to the mitochondrial mRNAs, rRNAs and, stained cytosolic 28S and 18S rRNAs as loading control.

Given the localization of LACTB2 in the mitochondria and its activity as an endoribonuclease, we hypothesized that LACTB2 plays a role in mitochondrial RNA metabolism. Therefore, we next asked if decreased expression of this protein can result in changes in the accumulation of all or a subset of mitochondrial transcripts. Decreased expression of LACTB2 was obtained by synthetic siRNA transfection to HEK-293E cells and then confirmed by qRT-PCR performed 48 h post transfection (Figure 8); LACTB2 protein level was reduced to ∼20% of that of the wild-type. We noticed, however, that the growth medium of the cells in which LACTB2 was reduced to ∼20% turned yellow, presumably because of changes in the glycolytic metabolism due to mitochondrial dysfunction (Figure 8B). This result is reminiscent of the effect of decreased expression of other important mitochondrial proteins (15,82,83). In addition, microscopic examination of the siRNA-treated cells disclosed dramatic morphologic changes in the shape and viability of the cells (Supplementary Figure S5). Together, these results demonstrated that normal LACTB2 expression is important for mitochondrial functioning and cell viability. However, the timescale of 48 h post-transfection is much shorter compared to that obtained when other proteins, that are essential for the function of the mitochondria, were down-expressed (15,82,83). In the case of LACTB2, analysis of longer time following the transfection, such as 72 h, could not be performed since most of the cells were already dead (not shown). The reason to this rapid lethal effect of the down-expression of LACTB2 waits further investigation. We next analyzed the effect of decreased expression on mitochondrial transcript accumulation in the mitochondria.

### Decreased expression of LACTB2 results in modest accumulation of mitochondrial transcripts

A modest accumulation of mitochondrial transcripts was observed in LACTB2-depleted cells compared to control cells transfected with scrambled RNA (Figure 8C). Mitochondrial mRNA accumulation ranged between none (Cox3) to ∼1.5–2.0-fold (ATP8, Cox2 and CYTB) over the levels seen in untreated cells. Accumulations of ∼1.5-fold were observed for ND2, ND3, ND4, ND4L, ND5 and ND6 transcripts. Small fluctuations in mitochondrial rRNAs and tRNAs levels, within the range of the standard error, were observed (Figure 8C). Finally, a 90% reduction in the nucleus-encoded transcript of LACTB2, was measured. While qRT-PCR is an excellent tool to determine the quantity of transcripts, it does not indicate the proportion of the full-length and correctly processed transcripts. RNA blotting was used to measure the accumulation and/or processing of full-length mitochondrial RNA transcripts. No significant differences were observed between the size and amount of transcripts in the cells containing only 20% of LACTB2 compared to the wild-type (Figure 8D), which seemed at odds with the differences seen in qPCR. It is likely that the quantitation of bands in RNA blots is not accurate enough to observe the 1.5–2-fold differences. As expected from an RNase, the hypothesis is that its decreased expression would result in the accumulation of the transcript(s) that is/are its initial target(s). Nevertheless, this modulation or another yet unidentified effect of the decreased expression caused the dysfunction of the mitochondria and damaged cell morphology. Similar effects of limited modulation of mitochondrial transcripts with significant mitochondria mis-functioning have been reported for the down regulation of the mitochondrial RNA-binding proteins LRPPRC and GRSF1 (15,25).

Upon overexpression of LACTB2 in HeLa cells (Supplementary Figure S6A), only a modest reduction of five mitochondrial mRNA transcripts was observed. Similar to the effect of decreased expression, the cell morphology became quiescent (Supplementary Figure S6B). It should be noted however, that microscopic examination of the GFP-LACTB2 overexpression in these cells revealed a significant mislocalization to the cytosol (not shown). This could contribute or being the cause for the cell defect upon overexpression. Because the protein is an endoribonuclease and exists as a monomer in the mitochondria, we proposed that LACTB2 plays an important role in mitochondrial gene expression. However, as with in other mitochondrial proteins involved in RNA metabolism, decreased expression of the protein and an analysis of the accumulation of mitochondrial transcripts failed to reveal its initial target (15,23,25).

## DISCUSSION

In a search for ribonucleases that are responsible for RNA degradation in human mitochondria, an uncharacterized protein from the MBL superfamily was identified. In this study, we report that the previously unannotated protein, LACTB2, displays endoribonuclease activity. Endogenous LACTB2 localized in the human mitochondrial matrix and existed as a soluble monomeric protein. Unlike other mitochondrial proteins, such as GRSF1 and RNase P (15) or PNPase and hSuv3 (14) that form protein complexes, no higher-order complexes of LACTB2 could be detected under our experimental conditions. However, it could not be ruled out that LACTB2 forms transient interactions with other mitochondrial proteins.

### Possible function of LACTB2 as an endoribonuclease in the mitochondria

Both strands of the mitochondrial 16K-base circular DNA are transcribed as polycistronic transcripts. The mRNAs are then derived from the polycistronic RNAs by cleavage of the ‘punctuating’ tRNAs via the action of the enzymes RNase P and RNase Z (ELAC2). Therefore, it appears that for the major mitochondrial endoribonucleolytic RNA processing events, an additional endoribonuclease is not necessary. However, there are three additional cleavages sites that are not coupled with tRNA processing. These include the generation of the 5΄ end of CoxI, the generation of the 3΄ end of ATP6 and the 5΄ end of CoxIII by cleavage of the di-cistronic transcript of these genes and the generation of the 5΄ end of Cyt B. An additional previously detected cleavage site on the 3΄ end of the ND6 transcript, the unique mRNA transcribed from the light strand, is questionable, since this transcript harbors a long 3΄ UTR (7). Although the generation of the CoxI and Cyt B 5΄ ends requires the vicinity of antisense sequences of tRNAs encoded by the light DNA strand, which can therefore potentially form a tRNA structure and be cleaved by the RNase P and Z, endonuclease cleavage activity is possible as well. Therefore, one potential function of LACTB2 could be the cleavage of these sites during the processing of the primary mitochondrial transcripts. However, this hypothesis is not supported by the equal accumulation of these transcripts in cells with decreased LACTB2 expression. Nevertheless, as discussed below, it is possible that another enzyme can compensate for this activity when LACTB2 is absent or that the remaining amount of LACTB2 is sufficient to maintain this activity.

In addition to the stable decoration of the 3΄ ends of mitochondrial mRNAs and rRNAs with poly(A)-tails, poly(A)-assisted degradation pathway takes place in this organelle as well (7). In bacteria and chloroplasts, this pathway is believed to be initiated by an endonucleolytic cleavage inside the transcript, followed by the addition of a transient poly(A)-tail serving as a platform for an exoribonuclease that further degrades the RNA (8). The poly(A)-polymerase (mtPAPI) and the exoribonuclease PNPase have already been described in human mitochondria and are most likely the major participants of this RNA degradation pathway (14,20). LACTB2 may function as the endoribonuclease that performs the endonucleolytic cleavage that initiates the degradation process, which is believed to be the rate-limiting step. The general modest accumulation of mitochondrial transcripts in LACTB2-deficient cells supports this hypothesis.

In our experiments aimed to characterize its biological function, increased or decreased LACTB2 expression resulted in a moderate but noticeable alterations in mitochondrial transcript levels. As expected from an RNase, the decreased expression of LACTB2 resulted in the accumulation of most tested RNA transcripts by 1.5–2.0-fold, and its overexpression caused a reduction to approximately half of that accumulated in control cells. These two mirrored observations generally observed with most of the tested transcripts, are compatible but were too moderate to enable identification of specific initial cleavage site(s). Similar non-specific, moderate and limited effects on the accumulation of mitochondrial transcripts were previously obtained with other RNA-mediated degradation proteins in mammalian mitochondria. For example, hPNPase-Suv3, PED12 and REXO2 demonstrated similar behavior and presented a challenge to identify their specific targets when downregulated in cell culture (14,22,23). Overall, none of these enzymes have been demonstrated to lead to significantly higher accumulation of certain mitochondrial transcripts upon their downregulation. This is also true for the mitochondria RNA binding proteins LRPPRC/SLIRP and GRSF1 (excluding COXI and II), which presented a modest reduction in the accumulation of mitochondrial transcripts upon downregulation, similar to LACTB2 (15,20). Recently, a novel RNA binding protein, FASTK, that primarily binds ND6 mRNA and is responsible for its biogenesis, was located in mitochondrial RNA granules (13). One possible explanation of the moderate effect of the manipulation of LACTB2 expression and the accumulation and processing of mitochondrial transcripts is that other ribonucleases, either those that are already known or those that have yet to be discovered, can compensate for its function. Despite moderate changes in the accumulation of mitochondria transcripts, manipulation of LACTB2 expression levels resulted in cellular morphological deformation and cell death, indicating that this protein is essential for mitochondria functioning and cell vitality. Moreover, the acidification of the medium and the cell death occurred much faster, in 2 days as compared to 4–6 days happened when other proteins that are involved in mitochondrial gene expression were down-expressed (15,82,83). This difference suggests a possible additional function of this protein, which is important for mitochondrial functioning and cell viability. Experiments to explore this possibility are underway.

### An MBL endoribonuclease in mammalian mitochondria

Members of the MBL superfamily that are ribonucleases are already well known and defined. Perhaps the most familiar ribonuclease in human cells is CPSF73, which is responsible for the endonucleolytic cleavage that, during transcription, defines the 3΄ end of nuclear-encoded mRNAs and the site of addition of the stable poly(A)-tail (40). Indeed, as described above, the structural homology of the catalytic active site of CPSF73 to that predicted for LACTB2, was a central factor in outlining the hypothesis that LACTB2 is a ribonuclease. An additional MBL member that is well known as a ribonuclease is RNase J. RNase J was discovered in *B*.*subtilis* as a functional replacement of RNase E, an mRNA endoribonuclease that is a major player in RNA degradation in *E. coli* (36,37,84). RNase J was then found to be present and potentially responsible for mRNA degradation in many bacteria, archaea and the chloroplasts of higher plants and green algae (which evolutionarily evolved from progenitors of cyanobacteria) (39,42,80,84). Interestingly, we previously showed that in a group of hyperthermophiles Methanogenic archaea, several members of the MBL superfamily are the sole homologs of known bacteria RNA-degrading enzymes, two of which are active as exo- and one as an endo-ribonuclease (42). These results suggest that RNA degradation is exclusively carried out in these archaea by MBL ribonucleases and indicate the evolutionary antiquity of these proteins as ribonucleases. Unlike plants, there is no RNase E homolog in mammalian genomes, and as mitochondria were evolutionarily derived from a progenitor α-proteobacteria, whose homologs today contain RNase J, it is perhaps not surprising that in addition to the specific RNase Z, an additional member of this family functions as a more general ribonuclease in mitochondria.

In contrast to CPSF73 and RNase J, but similar to RNase Z (ELAC2), LACTB2 contains the MBL domain but lacks the β-CASP and RMM domains; instead, LACTB2 has a C-terminal domain with no homology to other MBL proteins (Figure 2). A structural comparison of the active site interface demonstrated a shallow catalytic site cleft in LACTB2 and a deeper pocket for CPSF-73 and RNase J1 in *T. thermophiles* (Figure 3A and Supplementary Figure S1C). This shallow cleft interface may explain the broader substrate selection of LACTB2 compared to the specific assortment demonstrated by CPSF-73 and RNase J. This opened structure, as well as the observation that, unlike CPSF73, LACTB2 is not engaged with other proteins in a multi-protein complex, suggests that this protein is not specific to well-defined sequences or structures of the RNA substrate.

Our results show that human LACTB2 is an endoribonuclease, similar to other MBL members, such as the human CPSF73 and the archaeal mjRNase J2 (40,42). The absence of mononucleotide accumulation led to the conclusion that LACTB2 is neither an exonuclease nor a processive enzyme. Therefore, unlike the bacterial and chloroplast RNase J or the mammalian CPSF-73, LACTB2 does not possess dual endo- and exo- 5΄ to 3΄ ribonucleolytic activities.

### Changing certain amino acids confirmed the ribonucleolytic active site and indicated an additional RNA binding domain

Mapping the ribonucleolytic active and the RNA-binding sites of LACTB2 was achieved by mutating key residues of the protein. MBL mutations in motifs II and IV, which are involved in the coordination or bridging of the Zn^2+^ ions, confirmed that the histidine and aspartic acid motifs are essential for the catalytic and the RNA-binding activities, as seen with other MBL ribonucleases (37,40,42,68,72); the one exception is the mutation of D81 to Ala, which retains RNA-binding but not catalytic activity. Mutational analysis also suggests an elongating RNA-binding groove, with residues outside the catalytic site contributing to substrate binding and catalytic efficiency. Such a contribution of distal residues has recently been observed also in DNA repair exonucleases of the MBL family, suggesting that this is a general feature.

In conclusion, we identified a novel human mitochondrial protein, LACTB2, which functions as an endoribonuclease with a preference for cleavage 3΄ to purine-pyrimidine dinucleotide sequences of ssRNA substrates, while using Zn^2+^ ions within its catalytic site. Manipulations of LACTB2 protein expression hindered the mitochondrial functioning, resulting in changes in cell morphology and cell death. Unraveling the specific function of LACTB2 in mitochondrial RNA metabolism still requires further studies that will examine whether LACTB2 co-localizes with RNA-granules, identify its primary and perhaps specific, substrate *in vivo* and reveal the mechanism for the mitochondrial dysfunction when this protein is downregulated.

## Supplementary Material

Supplementary Data

SUPPLEMENTARY DATA

## ACCESSION NUMBERS

PDB ID : 4AD9
