# Supplementary material for: Identification of LACTB2, a metallo-β-lactamase protein, as a human mitochondrial endoribonuclease
Source: Nucleic Acids Res. 2016 Jan 29;44(4):1813–32. doi: 10.1093/nar/gkw050 (PMC4770246; doi:10.1093/nar/gkw050)
Supplement: Supplementary Data [file gkw050_Supp.zip › nar-01957-z-2015-File003.pdf]

Supplementary Information

Identification of LACTB2, a metallo-β-lactamase protein, as a human mitochondrial endoribonuclease

Shiri Levy[1], Charles K. Allerston[2,3], Varda Liveanu[1], Mouna H. Rohana[1], Opher Gileadi[2,*] and Gadi Schuster[1,*]

[1]Faculty of Biology, Technion- Israel Institute of Technology, Haifa 32000, Israel

[2]Structural Genomics Consortium, University of Oxford, Oxford OX3 7DQ, United Kingdom

[3] Current address: Charles.allerston@gmail.com

*To whom correspondence should be addressed. Tel: +972-4-8293171; Email: gadis@tx.technion.ac.il
Correspondence may also be addressed to O.G., Tel. +44-1865-617572; Email: opher.gileadi@sgc.ox.ac.uk

Supplemented Figures

Figure S1. LACTB2 active site exhibits high similarity to MBL ribonucleases RNase J1 and tRNase Z

A. The structure of the human LACTB2 active site (displayed in beige) is similar to that of the endo/exo ribonuclease RNase J1 (displayed in turquoise). Human LACTB2 (PDB 4AD9) and *T. thermophilus* RNase J (PDB:3BK2) were compared to draw structural similarity using the UCSF Chimera tool. The numbers of the homologous residues from LACTB2 (shown in the figure) and RNase J1 are, respectively, Motif II: His 77/75, His 79/77, Asp 81/79, and His 82/80. Motif III: His 145/150. Motif IV: Asp 164/172, and motif C/V: His 199/398. The two zinc ions are presented as purple spheres and hydrogen bonds are depicted as purple dashed lines. The single red bead represents a water or hydroxide oxygen.

B. The structure of the human LACTB2 (beige) active site is similar to that of the endoribonuclease tRNase Z (turquoise). Human LACTB2 (PDB:4AD9) and human nuclear tRNase Z (ELAC1; PDB:3ZWF) were compared to draw structural similarity using the UCSF Chimera tool. Residue numbers of LACTB2 (shown in the figure) and tRNase Z are, respectively, Motif II: His 77/62, His 79/64, Asp 81/66, and His 82/67. Motif III: His 145/182. Motif IV: Asp 164/253, and motif C/V: His 199/313.

C. The active site interface of human LACTB2 (4AD9) has a shallow catalytic cleft compared to a deeper pocket found in human CPSF73 (2I7T) and bacterial RNase J1 (3T3O). Ribbons represent the backbone of the proteins and the two light blue spheres represent the zinc ions. The structure of RNase J1 also includes a bound oligonucleotide, shown in a stick model.

Figure S2: LACTB2 cleaves $(AC)_{12}$ RNA substrate following C.

A 24 nt RNA oligo ribonucleotide with the sequence $(AC)_{12}$, radioactively labeled at the 5’ end, was incubated with the enzymes indicated. Incubation with LACTB2 was carried out for 15, 30 and 60 min at 37°C in buffer containing 12.5 mM Tris-HCl pH=7.4, 125 mM NaCl, 2.5 % glycerol and 0.25 mM DTT, as described in the materials and methods section. Incubation with RNase A (50 ng/ml) was performed on ice in the same buffer for 1, 2.5 and 5 min with the addition of 0.5 mg/ml yeast tRNA. Incubation with RNase P1 (0.01 u/ml) was performed as described for RNase A but in a buffer containing 20 mM Sodium acetate (pH=5.0), 0.5 mM Zinc acetate and 12% glycerol (1) . A 10 nt ladder is shown in the first lane to the left and an alkaline hydrolysis ladder of the $(AC)_{12}$ RNA is shown in the lane marked OH^-. NP: incubation of the RNA with no protein. Note that the $(AC)_{12}$ RNA is sensitive to hydrolysis when placed in a solution even for a short time and during the elution of the full length RNA from the gel (Lane labeled NP and data not shown). No difference in the migration of the cleavage products that have OH or phosphate group at the 3’ end is observed in this gel system. The only observed differences were in the migration of the cleavage products of RNase P1 that are shorter than 5 nt and slightly between the OH- and the other lanes. These differences could be attributed to the different salt concentrations. Indeed, no differences in the migration pattern were observed when the RNA were EtOH precipitated before loading on the gel (not shown).

Figure S3: The ribonucleolytic activity of LACTB2 is inhibited by EDTA and *o*-phenanthroline.

LACTB2 (7.5 pmol) was pre-incubated for 20 min with imidazole (50 mM), EDTA (50 mM) or *o*-phenanthroline (20 mM) and then a Cy3 5’ labeled 37 nt RNA substrate (10 pmol) was added and the incubation continued for 30 and 60 min, when RNA was purified and analyzed by PAGE. (-): RNA was incubated for 60 min with no addition of protein.

Figure S4. Mutated LACTB2 proteins display different RNA binding affinities.

A. The non-mutated (WT) and mutated versions of LACTB2 were incubated with [^{32}P]UTP-labeled RNA, followed by UV crosslinking, RNase A digestion, SDS-PAGE fractionation and autoradiography. NP- a control sample, in which no protein was added to the reaction mixture. A silver stained gel showing equal loading of the proteins is shown below the autoradiogram. In the lane labeled ∆motif II+WT, equal amounts of the two proteins were added to the reaction. Attempts to analyze the binding properties using a mobility shift assay without crosslinking were unsuccessful.

B. Electrostatic surface structure of LACTB2 (left), with a cartoon representation shown at the same orientation (right). Red and blue indicate negative and positive potentials (-/+ 5kT/e), respectively. The dotted black line indicates a putative trajectory of the RNA substrate. The sites of mutations tested in this study are shown on the cartoon model; mutations of R220, H216 and R217, which are distant from the catalytic site (motif II), severely reduce nuclease activity.

Figure S5. Effects of down-regulation of LACTB2 on cell morphology.

Image of HEK293 cells displaying morphological changes following 48h of LACTB2 down-regulation, as detected using a phase contrast cell observer microscope, with 60-fold magnification.

Figure S6. Effects of overexpression of human LACTB2 on mitochondrial transcript levels and on cell morphology.

Overexpression of LACTB2 causes a moderate decrease in the concentration of mitochondrial transcripts. A LACTB2 overexpression plasmid (pink bars) or an empty vector (brown bars) were transfected into HeLa cells. Cells were collected and analyzed 48h post-transfection by qRT-PCR. The GAPDH transcript was used as the reference gene for normalized expression calculations. The error bars show the standard error of the mean. **B.** Image of HeLa cells following 48h of LACTB2 overexpression, as detected under a phase contrast cell observer microscope, with 60-fold magnification.

REFERENCES CITED

1. Sollner-Webb, B., Cruz-Reyes, J. and Rusche, L.N. (2001) Direct sizing of RNA fragments using RNase-generated standards. *Methods Enzymol*, **342**, 378-383.

Figure S1.

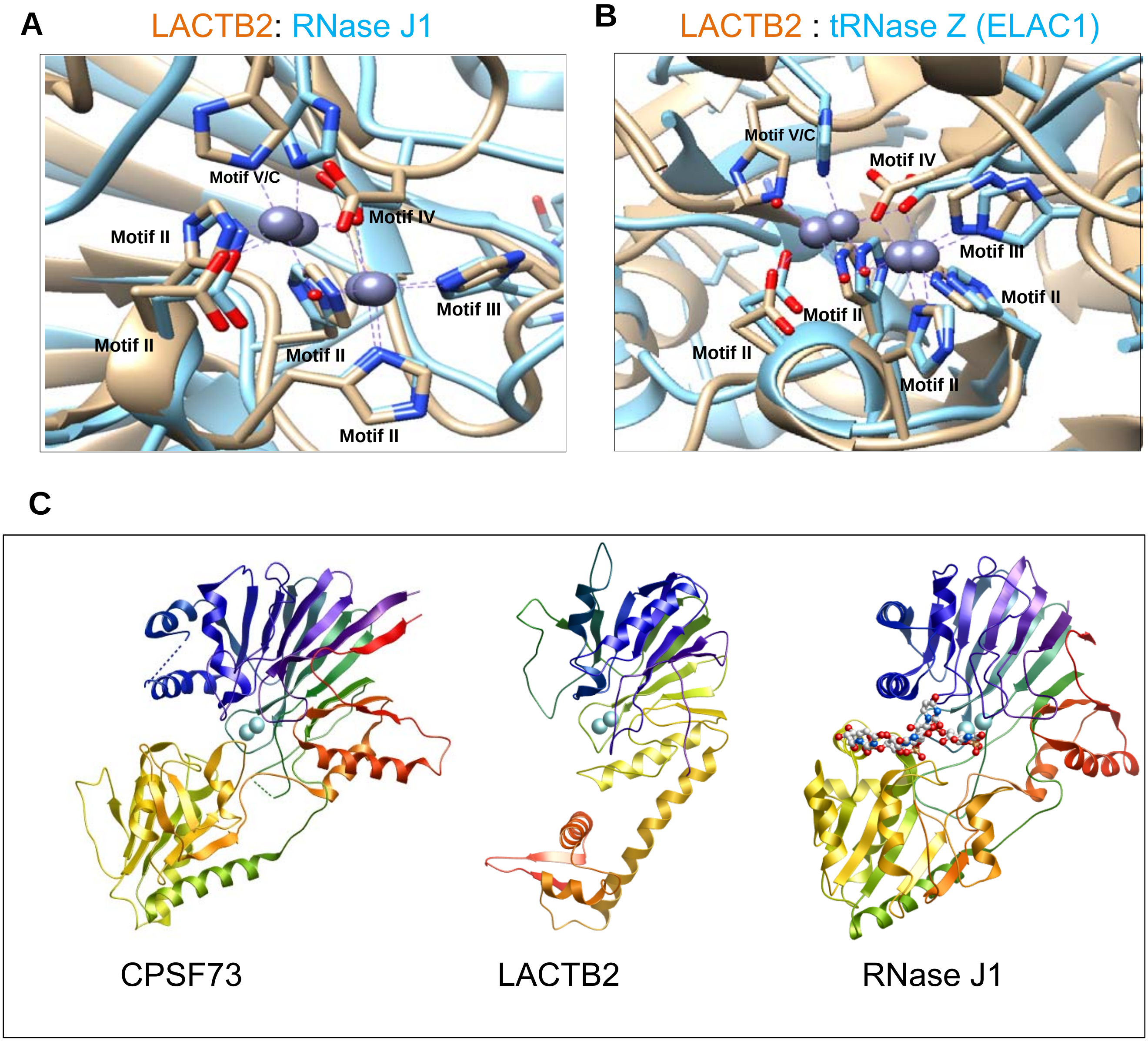
A
LACTB2: RNase J1
Motif V/C
Motif II
Motif IV
Motif III
Motif II
Motif II
Motif II
B
LACTB2 : tRNase Z (ELAC1)
Motif V/C
Motif IV
Motif III
Motif II
Motif II
Motif II
Motif II
C
CPSF73
LACTB2
RNase J1

Figure S2

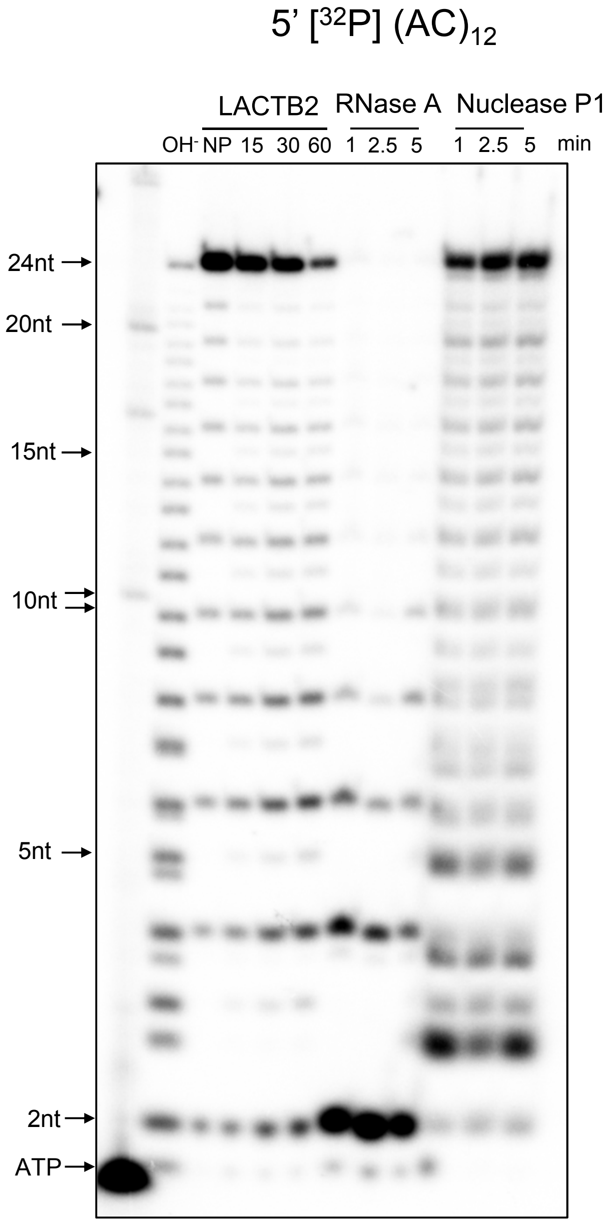
5' [32P] (AC)12
LACTB2
RNase A
Nuclease P1
OH- NP 15 30 60 1 2.5 5 1 2.5 5 min
24nt
20nt
15nt
10nt
5nt
2nt
ATP

Figure S3.

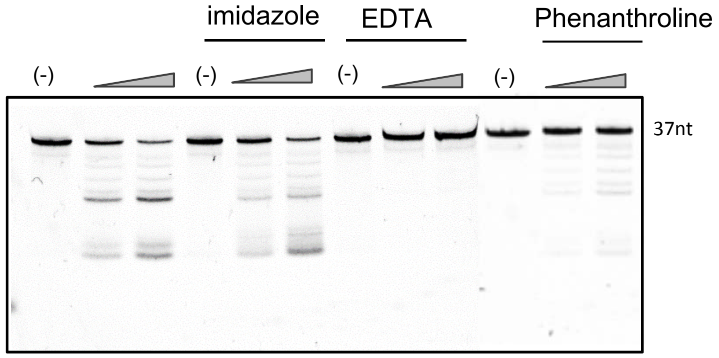

Figure S4.

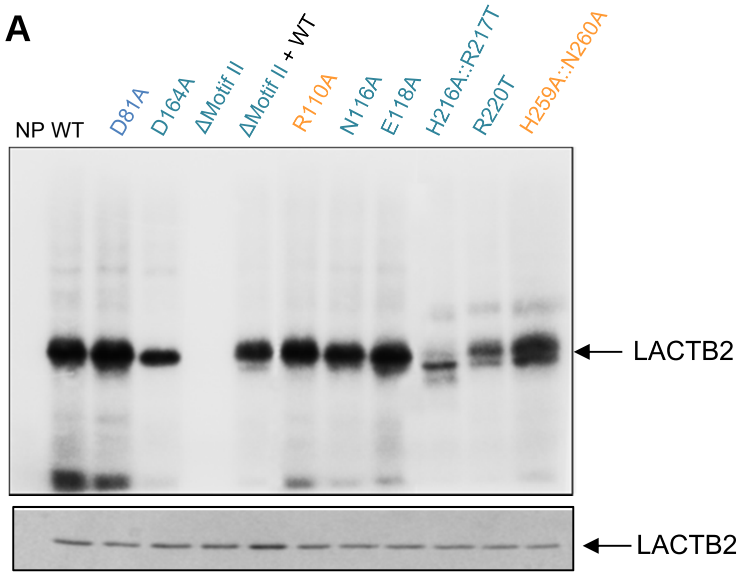
A
NP
WT
D81A
D164A
ΔMotif II
ΔMotif II + WT
R110A
N116A
E118A
H216A::R217T
R220T
H259A::N260A
LACTB2
LACTB2

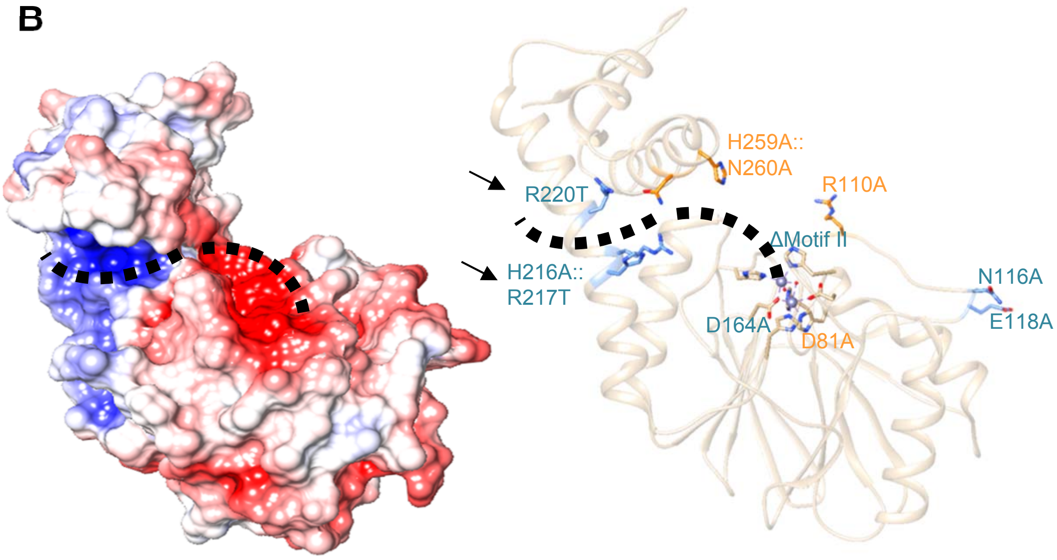
B
H259A::
N260A
R220T
R110A
ΔMotif II
H216A::
R217T
N116A
E118A
D164A
D81A

Figure S5.

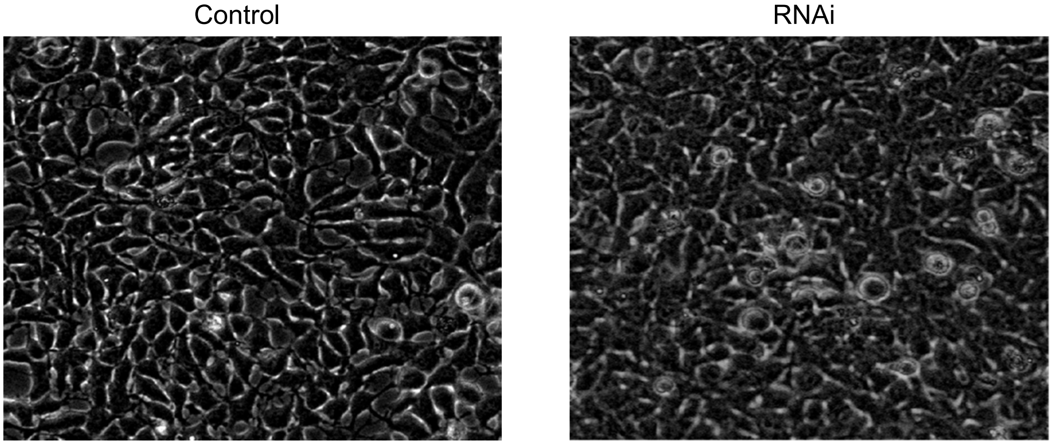

Figure S6.

A

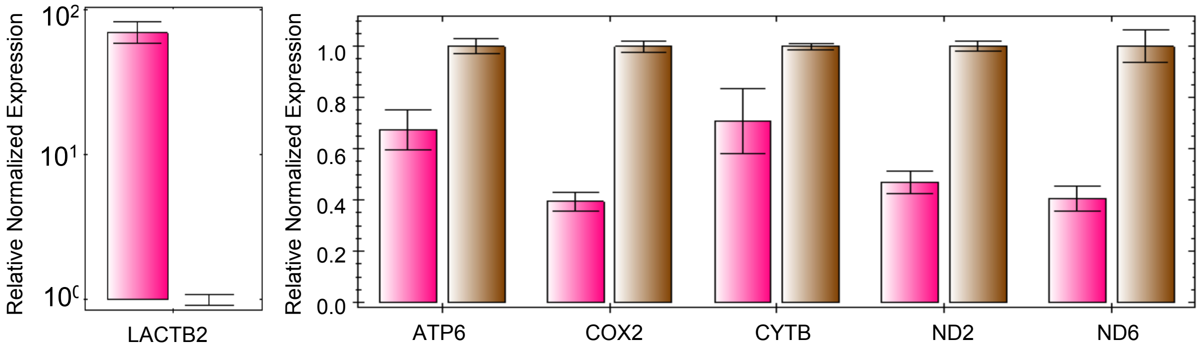

Relative Normalized Expression
LACTB2
ATP6
COX2
CYTB
ND2
ND6

B

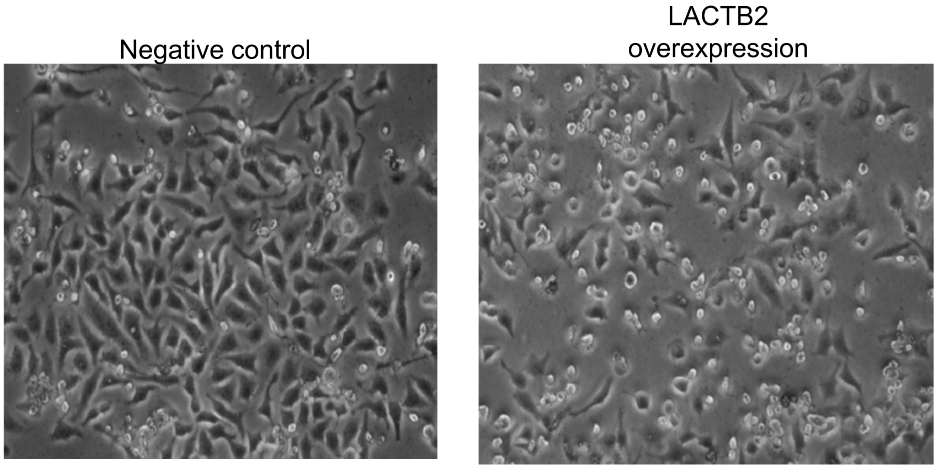

Negative control
LACTB2 overexpression

Supplemented Tables

Table S1: Oligonucleotide primers used for cloning and site directed mutagenesis

Primer name	Sequence 5’-3’
LACTB2_F	TACTTCCAATCCATGGCTGCTGTACTGCAGCG
LACTB2_R	TATCCACCTTTACTGTCAAAGATGAGCTTTCCATTTCTTGTC
Δ motif II_F	CCAGGAAATTGTAGTGACTTCTGGAGGCATAGGAGATATTTGTA
Δ motif II_R	TACAAATATCTCCTATGCCTCCAGAAGTCACTACAATTTCCTGG
D81A_F	CTCACTGGCACCGAGCTCATTCTGGAGGCAT
D81A_R	ATGCCTCCAGAATGAGCTCGGTGCCAGTGAG
D164A_F	TGCTATCTTTTCTGGAGCTTGCATCCTAGGGGAAG
D164A_R	CTTCCCCTAGGATGCAAGCTCCAGAAAAGATAGCA
R110A_F	AACTCCCACGGAATCCTCAGGCAGAAGAAATTATAGGAAATGG
R110A_R	CCATTTCCTATAATTTCTTCTGCCTGAGGATTCCGTGGGAGTT
N116A_F	CGGAATCCTCAGAGAGAAGAAATTATAGGAGCTGGAGAGCAACAATAT
N116A_R	ATATTGTTGCTCTCCAGCTCCTATAATTTCTTCTCTCTGAGGATTCCG
E118A_F	GAGAAGAAATTATAGGAAATGGAGCGCAACAATATGTTTATCTGAAAGA
E118A_R	TCTTTCAGATAAACATATTGTTGCGCTCCATTTCCTATAATTTCTTCTC
H216A::R217T_F	ATGCTGAAGCTAAAATTCAACAATACATTTCTGCCACGAATATTCGAGAGCAGCAAATT CTTAC
H216A::R217T_R	GTAAGAATTTGCTGCTCTCGAATATTCGTGGCAGAAATGTATTGTTGAATTTTAGCTTCA GCAT
R220T_F	GAAGCTAAAATTCAACAATACATTTCTCACAGAAATATTACGGAGCAGCAAATTCTTACA TTATTTCG
R220T_R	CGAAATAATGTAAGAATTTGCTGCTCCGTAATATTTCTGTGAGAAATGTATTGTTGAATT TTAGCTTC
H259::N260A_F	ACAAGAATACTCCTGAGAATTTACATGAAATGGCTAAAGCTGCTCTCTTACTTCATTTGA AAAAACTAG
H259::N260A_R	CTAGTTTTTTCAAATGAAGTAAGAGAGCAGCTTTAGCCATTTCATGTAAATTCTCAGGAG TATTCTTGT

Table S2: Synthetic RNA oligonucleotide used in this work

**RNA**	**Sequence 5’-3’**
30nt	AAUUUCUAAUAAUUAAUACAUUUUUCCUCU
37nt	AAUUUCUAAUAAUUAAUAUGUUUUUCCUCUCACACAC
40nt	GGGAAUUUCUAAUAAUUAAUACAUUUUUCCUCUCACACAC
Poly $(A)_{20}$	AAAAAAAAAAAAAAAAAAAA
Poly $(U)_{20}$	UUUUUUUUUUUUUUUUUUUU
Poly $(GU)_{12}$	GUGUGUGUGUGUGUGUGUGUGUGU
Poly $(AC)_{12}$	ACACACACACACACACACACACAC
16nt linear	UCUAAUAUAAUUAAU
30nt stem loop	UCUAAUAUAAUUAAUAUUAAUUAUAUUAGA

Table S3: Oligonucleotide primers used for *in vitro* transcription of the 40nt RNA

**DNA**	**Sequence 5’-3’**
40nt F	TAATACGACTCACTATAGGGAATTTCTAATAATTAATACATTTTTCCTCTCTCACACAC
40nt R	ATTATGCTGAGTGATATCCCTTAAAGATTATTAATTATGTAAAAAGGAGAGAGTGTGTG

Table S4: siRNA duplexes used for down expression of LACTB2

**Duplex name**	**Sequence**
LACTB2 sense<br>LACTB2 antisense	5’-ACAGCUGAUGUAUUCUGGAAUUGCUGG-3’<br>5’-TGUCGACUACAUAAGACCUUAACGA-3’
Negative control sense<br>Negative control antisense	5’-CGUUAAUCGCGUAUAAUACGCGUAT-3’<br>3’-AUACGCGUAUUAUACGCGAUUAACGAC-5’

Table S5: Oligonucleotide used for the detection of transcripts by Northern Blot analysis

**Primer name**	**Sequence 5’-3’**
NB LACTB2	GATGCTTTTACAAATATCTCCTATGCCTCCAGAATGATCTCGGTGCCAGT
NB GAPDH	GAAGACGCCAGTGGACTCCACGACGTACTCAGCGCCAGCATCGCCCCACT
NB ND2	GATTGAGAGAGTGAGGAGAAGGCTTACGTTTAGTGAGGGAGAGATTTGGT
NB ND5	GCTGCCAATGGTGAGGGAGGTTGAAGTGAGAGGTATGGTTTTGAGTAGTC
NB ND6	GCTAACCCCACTAAAACACTCACCAAGACCTCAACCCCTGACCCCCATGC
NB COX1	GTTTGCTAATACAATGCCAGTCAGGCCACCTACGGTGAAAAGAAAGATGA
NB COX2	GGATGCGTAGGGATGGGAGGGCGATGAGGACTAGGATGATGGCGGGCAGG
NB ATP6	TATAGATAGTTGGGTGGTTGGTGTAAATGAGTGAGGCAGGAGTCCGAGGA
NB ATP8	CTTTGGTGAGGGAGGTAGGTGGTAGTTTGTGTTTAATATTTTTAGTTGGG

NB 12S	GTTTTAAGCTGTGGCTCGTAGTGTTCTGGCGAGCAGTTTTGTTGATTTAA
NB ND1	TATAAGTAATGCTAGGGTGAGTGGTAGGAAGTTTTTTCATAGGAGGTGTA
NB ND3	GTGGCAGGTTAGTTGTTTGTAGGGCTCATGGTAGGGGTAAAAGGAGGGCA
NB ND4	TGAGAATGACTGCGCCGGTGAAGCTTCAGGGGGTTTGGATGAGAATGGCT
NB ND4L	ACAATATTGGCTAAGAGGGAGTGGGTGTTGAGGGTTATGAGAGTAGCTAT
NB COX3	TGCGAGTAATACGGATGTGTTTAGGAGTGGGACTTCTAGGGGATTTAGCG
NB CYTB	AGGAGAGAAGGAAGAGAAGTAAGCCGAGGGCGTCTTTGATTGTGTAGTAA
NB 16S	TGAGGAGTAGGAGGTTGGCCATGGGTATGTTGTTAAGAAGAGGAATTGAA

Table S6: Oligonucleotide primers used for Real Time PCR analysis

Primer name	Sequence 5'-3'
LACTB2_F	CAAGGCACCAACACCTACCT
LACTB2_R	AGAATGATCTCGGTGCCAGT
GAPDH_F	ATCACCATCTTCCAGGAGCGA
GAPDH_R	TTCTCCATGGTGGTGAAGACG
12S_F	GCAAGCATCCCCGTTCCAGTG
12S_R	TTCCCGTGGGGGTGTGGCTA
16S_F	AAAGCGTTCAAGCTCAACACC
16S_R	TAATCTGACGCAGGCTTATGC
ND1_F	ACAACCCTTCGCTGACGCCAT
ND1_R	AGAGCTAAGGTCGGGGCGGT
ND2_F	TCCTCACGCAAGCAACCGCA
ND2_R	GCCGGATGTCAGAGGGGTGC
ND3_F	CCTATATCCCCCGCCCGCGT
ND3_R	GTTTGTAGGGCTCATGGTAGGGGT
ND4_F	CCTCCGACCCCCTAACAACCCCC
ND4_R	AGTGGCGTTGGCTTGCCATGA
ND4L_F	CGCTCACACCTCATATCCTCCCTAC
ND4L_R	AAGAGGGAGTGGGTGTTGAGGGTT
ND5_F	ATCGGTTTCATCCTCGCCTTA
ND5_R	ACCTAATTGGGCTGATTTGCC
ND6_F	AACCCCTGACCCCCATGCCTC
ND6_R	GCGGTGTGGTCGGGTGTGTTA
COX1_F	TCGGTGCCCCCGATATGGCGT
COX1_R	ACCTGTTCCTGCTCCGGCCTCCA
COX2_F	ACAGATGCAATTCCCGGACGT

COX2_R	GGCATGAAACTGTGGTTTGCT
COX3_F	AGCCACAGGCTTCCACGGACT
COX3_R	CCAGTATCAGGCGGCGGCTTCG
ATP6_F	GGCACACCTACACCCCTTATCCCCA
ATP6_R	AGCGGTTAGGCGTACGGCCAGG
ATP8_F	CCTACCTCCCTCACCAAAGCCCA
ATP8_R	TGTGGGGGCAATGAATGAAGCG
Cyt B_F	AGTCCCACCCTCACACGATTC
Cyt B_R	AGTAAGCCGAGGGCGTCTTTG
tRNA Val_F	CAGAGTGTAGCTTAACACAAAG
tRNA Val_R	TCAGAGCGGTCAAGTTAAGTT
tRNA Glu_F	TGTAGTTGAAATACAACGATGGT
tRNA Glu_F	TATTCTCGCACGGACTACAA
tRNA Tyr_F	GGTAAAATGGCTGAGTGAAGCA
tRNA Tyr_R	TGGTAAAAAGAGGCCTAACC
tRNA Lys_F	CACTGTAAAGCTAACTTAGCATTAA
tRNA Lys_R	TCACTGTAAAGAGGTGTTGGTTCTC
