# Supplementary material for: Identification of LACTB2, a metallo-β-lactamase protein, as a human mitochondrial endoribonuclease
Source: Nucleic Acids Res. 2016 Jan 29;44(4):1813–32. doi: 10.1093/nar/gkw050 (PMC4770246; doi:10.1093/nar/gkw050)
Supplement: Supplementary Data [file gkw050_Supp.zip › nar-01957-z-2015-File004.pdf]

Full wwPDB X-ray Structure Validation Report (i)

Feb 28, 2014 – 11:25 PM GMT

PDB ID	:	4AD9
Title	:	Crystal structure of human LACTB2.
Authors	:	Allerston, C.K.; Krojer, T.; Shrestha, B.; Burgess Brown, N.; Chalk, R.; Elkins, J.M.; Filippakopoulos, P.; Pike, A.C.W.; Muniz, J.R.C.; Vollmar, M.; Arrowsmith, C.H.; Weigelt, J.; Edwards, A.; Bountra, C.; Von Delft, F.; Gileadi, O.
Deposited on	:	2011-12-22
Resolution	:	2.60 Å(reported)

This is a full wwPDB validation report for a publicly released PDB entry.
We welcome your comments at validation@mail.wwpdb.org
A user guide is available at http://wwpdb.org/ValidationPDFNotes.html

---

The following versions of software and data (see references) were used in the production of this report:

MolProbity	:	4.02b-467
Mogul	:	1.15 2013
Xtriage (Phenix)	:	dev-1323
EDS	:	stable22639
Percentile statistics	:	21963
Refmac	:	5.8.0049
CCP4	:	6.3.0 (Settle)
Ideal geometry (proteins)	:	Engh & Huber (2001)
Ideal geometry (DNA, RNA)	:	Parkinson et. al. (1996)
Validation Pipeline (wwPDB-VP)	:	stable22683

1 Overall quality at a glance (i)

The reported resolution of this entry is 2.60 Å.

Percentile scores (ranging between 0-100) for global validation metrics of the entry are shown in the following graphic. The table shows the number of entries on which the scores are based.

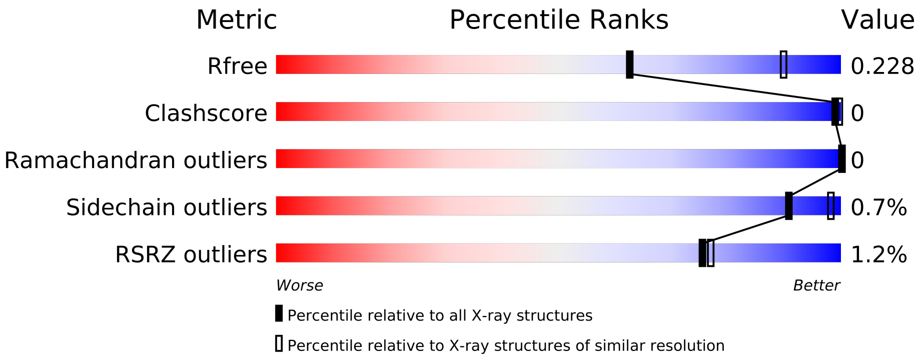

Metric	Whole archive (#Entries)	Similar resolution (#Entries, resolution range(Å))
R_{free}	66092	1718 (2.60-2.60)
Clashscore	79885	2154 (2.60-2.60)
Ramachandran outliers	78287	2113 (2.60-2.60)
Sidechain outliers	78261	2113 (2.60-2.60)
RSRZ outliers	66119	1718 (2.60-2.60)

The table below summarises the geometric issues observed across the polymeric chains and their fit to the electron density. The red, orange, yellow and green segments on the lower bar indicate the fraction of residues that contain outliers for >=3, 2, 1 and 0 types of geometric quality criteria. The upper red bar (where present) indicates the fraction of residues that have poor fit to the electron density.

Mol	Chain	Length	Quality of chain
1	A	289	
1	B	289	
1	C	289	
1	D	289	
1	E	289	
1	F	289	

The following table lists non-polymeric compounds that are outliers for geometric or electron-density-fit criteria:

Mol	Type	Chain	Res	Geometry	Electron density
3	EDO	A	1289	-	X
3	EDO	F	1289	-	X

2 Entry composition (i)

There are 4 unique types of molecules in this entry. The entry contains 14225 atoms, of which 0 are hydrogen and 0 are deuterium.

In the tables below, the ZeroOcc column contains the number of atoms modelled with zero occupancy, the AltConf column contains the number of residues with at least one atom in alternate conformation and the Trace column contains the number of residues modelled with at most 2 atoms.

- Molecule 1 is a protein called BETA-LACTAMASE-LIKE PROTEIN 2.

Mol	Chain	Residues	Atoms	ZeroOcc	AltConf	Trace
1	A	288	Total C N O S<br>2230 1423 387 410 10	0	0	0
1	B	285	Total C N O S<br>2104 1341 363 391 9	0	0	0
1	C	285	Total C N O S<br>2242 1424 391 417 10	0	1	0
1	D	288	Total C N O S<br>2235 1423 387 415 10	0	0	0
1	E	284	Total C N O S<br>2093 1331 362 391 9	0	0	0
1	F	285	Total C N O S<br>2238 1424 387 418 9	0	1	0

There are 6 discrepancies between the modelled and reference sequences:

Chain	Residue	Modelled	Actual	Comment	Reference
A	0	SER	-	EXPRESSION TAG	UNP Q53H82
B	0	SER	-	EXPRESSION TAG	UNP Q53H82
C	0	SER	-	EXPRESSION TAG	UNP Q53H82
D	0	SER	-	EXPRESSION TAG	UNP Q53H82
E	0	SER	-	EXPRESSION TAG	UNP Q53H82
F	0	SER	-	EXPRESSION TAG	UNP Q53H82

- Molecule 2 is ZINC ION (three-letter code: ZN) (formula: Zn).

Mol	Chain	Residues	Atoms	ZeroOcc	AltConf
2	D	2	Total Zn<br>2 2	0	0
2	E	2	Total Zn<br>2 2	0	0
2	B	2	Total Zn<br>2 2	0	0

Continued on next page...

Continued from previous page...

Mol	Chain	Residues	Atoms	ZeroOcc	AltConf
2	C	2	Total Zn<br>2 2	0	0
2	A	2	Total Zn<br>2 2	0	0
2	F	2	Total Zn<br>2 2	0	0

- Molecule 3 is 1,2-ETHANEDIOL (three-letter code: EDO) (formula: $C_2H_6O_2$).

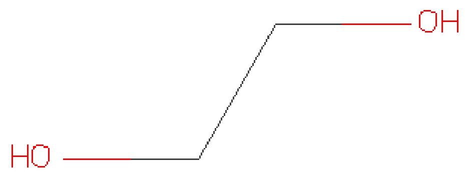

Mol	Chain	Residues	Atoms	ZeroOcc	AltConf
3	A	1	Total C O<br>4 2 2	0	0
3	D	1	Total C O<br>4 2 2	0	0
3	D	1	Total C O<br>4 2 2	0	0
3	F	1	Total C O<br>4 2 2	0	0

- Molecule 4 is water.

Mol	Chain	Residues	Atoms	ZeroOcc	AltConf
4	A	240	Total O<br>240 240	0	0
4	B	94	Total O<br>94 94	0	0

Continued on next page...

Continued from previous page...

Mol	Chain	Residues	Atoms	ZeroOcc	AltConf
4	C	205	Total O<br>205 205	0	0
4	D	205	Total O<br>205 205	0	0
4	E	97	Total O<br>97 97	0	0
4	F	214	Total O<br>214 214	0	0

3 Residue-property plots ⓘ

These plots are drawn for all protein, RNA and DNA chains in the entry. The first graphic for a chain summarises the proportions of errors displayed in the second graphic. The second graphic shows the sequence view annotated by issues in geometry and electron density. Residues are color-coded according to the number of geometric quality criteria for which they contain at least one outlier: green = 0, yellow = 1, orange = 2 and red = 3 or more. A red dot above a residue indicates a poor fit to the electron density (RSRZ > 2). Stretches of 2 or more consecutive residues without any outlier are shown as a green connector. Residues present in the sample, but not in the model, are shown in grey.

- Molecule 1: BETA-LACTAMASE-LIKE PROTEIN 2

Chain A:

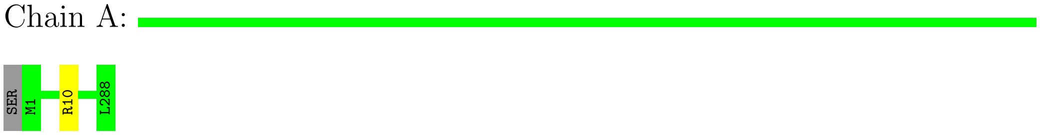

- Molecule 1: BETA-LACTAMASE-LIKE PROTEIN 2

Chain B:

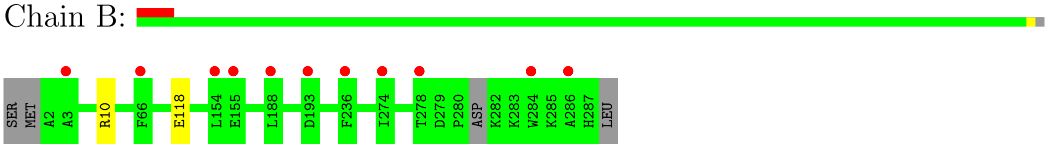

- Molecule 1: BETA-LACTAMASE-LIKE PROTEIN 2

Chain C:

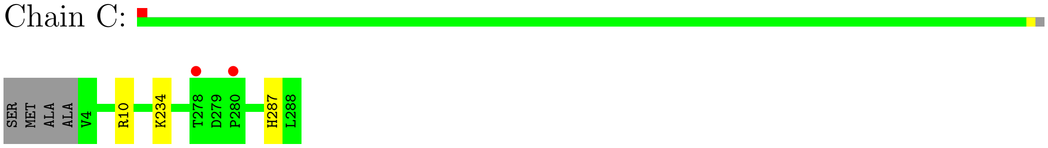

- Molecule 1: BETA-LACTAMASE-LIKE PROTEIN 2

Chain D:

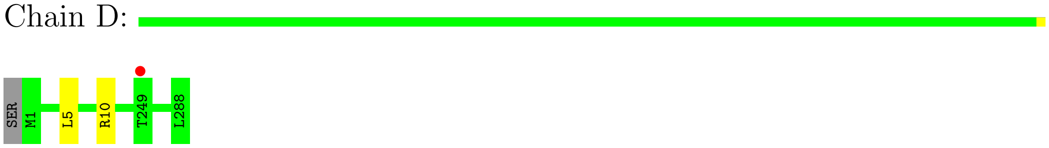

- Molecule 1: BETA-LACTAMASE-LIKE PROTEIN 2

Chain E:

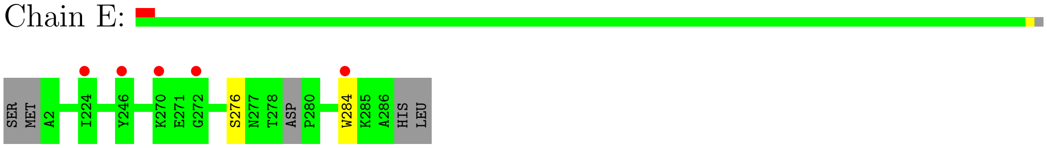

- Molecule 1: BETA-LACTAMASE-LIKE PROTEIN 2

Chain F:

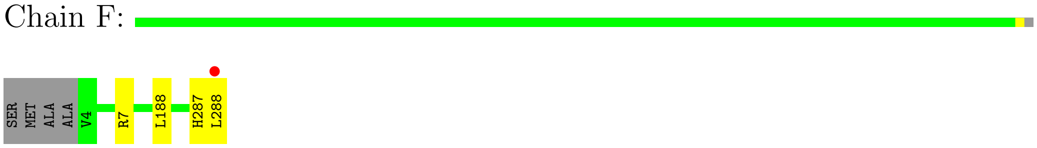

4 Data and refinement statistics ⓘ

Property	Value	Source
Space group	P 1 21 1	Depositor
Cell constants<br>a, b, c, α, β, γ	107.47Å 95.69Å 135.76Å<br>90.00° 112.84° 90.00°	Depositor
Resolution (Å)	46.85 – 2.60<br>46.85 – 2.60	Depositor<br>EDS
% Data completeness<br>(in resolution range)	99.2 (46.85-2.60)<br>99.2 (46.85-2.60)	Depositor<br>EDS
R_{merge}	0.07	Depositor
R_{sym}	(Not available)	Depositor
$< I/\sigma(I) >$ [1]	2.12 (at 2.61Å)	Xtriage
Refinement program	BUSTER 2.10.0	Depositor
R, R_{free}	0.177 , 0.224<br>0.184 , 0.228	Depositor<br>DCC
R_{free} test set	1957 reflections (2.59%)	DCC
Wilson B-factor (Å^2)	35.2	Xtriage
Anisotropy	0.415	Xtriage
Bulk solvent k_{sol}(e/Å^3), B_{sol}(Å^2)	0.33 , 48.2	EDS
Estimated twinning fraction	0.019 for h,-k,-h-l	Xtriage
L-test for twinning	$< \vert L \vert > = 0.49$, $< L^2 > = 0.32$	Xtriage
Outliers	0 of 77582 reflections	Xtriage
F_o,F_c correlation	0.94	EDS
Total number of atoms	14225	wwPDB-VP
Average B, all atoms (Å^2)	46.0	wwPDB-VP

Xtriage's analysis on translational NCS is as follows: *The largest off-origin peak in the Patterson function is 15.37% of the height of the origin peak. No significant pseudotranslation is detected.*

[1] Intensities estimated from amplitudes.

5 Model quality (i)

5.1 Standard geometry (i)

Bond lengths and bond angles in the following residue types are not validated in this section: ZN, EDO

The Z score for a bond length (or angle) is the number of standard deviations the observed value is removed from the expected value. A bond length (or angle) with $|Z| > 5$ is considered an outlier worth inspection. RMSZ is the root-mean-square of all Z scores of the bond lengths (or angles).

Mol	Chain	Bond lengths		Bond angles	
		RMSZ	#|Z| >5	RMSZ	#|Z| >5
1	A	0.54	0/2275	0.69	0/3088
1	B	0.43	0/2143	0.65	0/2920
1	C	0.52	0/2290	0.68	0/3108
1	D	0.52	0/2280	0.68	0/3096
1	E	0.44	0/2133	0.64	0/2907
1	F	0.54	0/2286	0.69	0/3101
All	All	0.50	0/13407	0.67	0/18220

There are no bond length outliers.

There are no bond angle outliers.

There are no chirality outliers.

There are no planarity outliers.

5.2 Close contacts (i)

In the following table, the Non-H and H(model) columns list the number of non-hydrogen atoms and hydrogen atoms in the chain respectively. The H(added) column lists the number of hydrogens added by MolProbity. The Clashes column lists the number of clashes within the asymmetric unit, and the number in parentheses is this value normalized per 1000 atoms of the molecule in the chain. The Symm-Clashes column gives symmetry related clashes, in the same way as for the Clashes column.

Mol	Chain	Non-H	H(model)	H(added)	Clashes	Symm-Clashes
1	A	2230	0	0	0	0
1	B	2104	0	0	1	0
1	C	2242	0	0	0	0
1	D	2235	0	0	0	0
1	E	2093	0	0	1	0
1	F	2238	0	0	1	0

Continued on next page...

Continued from previous page...

Mol	Chain	Non-H	H(model)	H(added)	Clashes	Symm-Clashes
2	A	2	0	0	0	0
2	B	2	0	0	0	0
2	C	2	0	0	0	0
2	D	2	0	0	0	0
2	E	2	0	0	0	0
2	F	2	0	0	0	0
3	A	4	0	6	0	0
3	D	8	0	12	0	0
3	F	4	0	6	0	0
4	A	240	0	0	0	0
4	B	94	0	0	1	0
4	C	205	0	0	0	0
4	D	205	0	0	0	0
4	E	97	0	0	0	0
4	F	214	0	0	0	0
All	All	14225	0	24	3	0

Clashscore is defined as the number of clashes calculated for the entry per 1000 atoms (including hydrogens) of the entry. The overall clashscore for this entry is 0.

All (3) close contacts within the same asymmetric unit are listed below.

Atom-1	Atom-2	Distance(Å)	Clash(Å)
1:F:287:HIS:O	1:F:288:LEU:CB	2.38	0.71
1:E:276:SER:OG	1:E:284:TRP:NE1	2.43	0.51
1:B:118:GLU:OE2	4:B:2064:HOH:O	2.21	0.42

There are no symmetry-related clashes.

5.3 Torsion angles

5.3.1 Protein backbone (i)

In the following table, the Percentiles column shows the percent Ramachandran outliers of the chain as a percentile score with respect to all X-ray entries followed by that with respect to entries of similar resolution. The Analysed column shows the number of residues for which the backbone conformation was analysed, and the total number of residues.

Mol	Chain	Analysed	Favoured	Allowed	Outliers	Percentiles	
1	A	286/289 (99%)	279 (98%)	7 (2%)	0	100	100
1	B	281/289 (97%)	274 (98%)	7 (2%)	0	100	100

Continued on next page...

Continued from previous page...

Mol	Chain	Analysed	Favoured	Allowed	Outliers	Percentiles	
1	C	284/289 (98%)	276 (97%)	8 (3%)	0	100	100
1	D	286/289 (99%)	278 (97%)	8 (3%)	0	100	100
1	E	280/289 (97%)	271 (97%)	9 (3%)	0	100	100
1	F	284/289 (98%)	275 (97%)	9 (3%)	0	100	100
All	All	1701/1734 (98%)	1653 (97%)	48 (3%)	0	100	100

There are no Ramachandran outliers to report.

5.3.2 Protein sidechains (i)

In the following table, the Percentiles column shows the percent sidechain outliers of the chain as a percentile score with respect to all X-ray entries followed by that with respect to entries of similar resolution. The Analysed column shows the number of residues for which the sidechain conformation was analysed, and the total number of residues.

Mol	Chain	Analysed	Rotameric	Outliers	Percentiles	
1	A	229/256 (90%)	228 (100%)	1 (0%)	95	99
1	B	200/256 (78%)	199 (100%)	1 (0%)	94	99
1	C	238/256 (93%)	235 (99%)	3 (1%)	80	95
1	D	232/256 (91%)	230 (99%)	2 (1%)	87	97
1	E	199/256 (78%)	199 (100%)	0	100	100
1	F	235/256 (92%)	233 (99%)	2 (1%)	87	97
All	All	1333/1536 (87%)	1324 (99%)	9 (1%)	91	98

All (9) residues with a non-rotameric sidechain are listed below:

Mol	Chain	Res	Type
1	A	10	ARG
1	B	10	ARG
1	C	10	ARG
1	C	234	LYS
1	C	287	HIS
1	D	5	LEU
1	D	10	ARG
1	F	7	ARG
1	F	188	LEU

Some sidechains can be flipped to improve hydrogen bonding and reduce clashes. There are no such sidechains identified.

5.3.3 RNA (i)

There are no RNA chains in this entry.

5.4 Non-standard residues in protein, DNA, RNA chains (i)

There are no non-standard protein/DNA/RNA residues in this entry.

5.5 Carbohydrates (i)

There are no carbohydrates in this entry.

5.6 Ligand geometry (i)

Of 16 ligands modelled in this entry, 12 are monoatomic - leaving 4 for Mogul analysis.

In the following table, the Counts columns list the number of bonds (or angles) for which Mogul statistics could be retrieved, the number of bonds (or angles) that are observed in the model and the number of bonds (or angles) that are defined in the chemical component dictionary. The Link column lists molecule types, if any, to which the group is linked. The Z score for a bond length (or angle) is the number of standard deviations the observed value is removed from the expected value. A bond length (or angle) with $|Z| > 2$ is considered an outlier worth inspection. RMSZ is the root-mean-square of all Z scores of the bond lengths (or angles).

Mol	Type	Chain	Res	Link	Bond lengths			Bond angles		
					Counts	RMSZ	$\#\|Z\| > 2$	Counts	RMSZ	$\#\|Z\| > 2$
3	EDO	A	1289	-	3,3,3	0.44	0	2,2,2	0.67	0
3	EDO	D	1289	-	3,3,3	0.45	0	2,2,2	0.62	0
3	EDO	D	1290	-	3,3,3	0.51	0	2,2,2	0.64	0
3	EDO	F	1289	-	3,3,3	0.60	0	2,2,2	0.31	0

In the following table, the Chirals column lists the number of chiral outliers, the number of chiral centers analysed, the number of these observed in the model and the number defined in the chemical component dictionary. Similar counts are reported in the Torsion and Rings columns. '-' means no outliers of that kind were identified.

Mol	Type	Chain	Res	Link	Chirals	Torsions	Rings
3	EDO	A	1289	-	-	0/1/1/1	0/0/0/0
3	EDO	D	1289	-	-	0/1/1/1	0/0/0/0
3	EDO	D	1290	-	-	0/1/1/1	0/0/0/0

Continued on next page...

Continued from previous page...

Mol	Type	Chain	Res	Link	Chirals	Torsions	Rings
3	EDO	F	1289	-	-	0/1/1/1	0/0/0/0

There are no bond length outliers.

There are no bond angle outliers.

There are no chirality outliers.

There are no torsion outliers.

There are no ring outliers.

5.7 Other polymers (i)

There are no such residues in this entry.

5.8 Polymer linkage issues

There are no chain breaks in this entry.

6 Fit of model and data (i)

6.1 Protein, DNA and RNA chains (i)

In the following table, the column labelled '#RSRZ> 2' contains the number (and percentage) of RSRZ outliers, followed by percent RSRZ outliers for the chain as percentile scores relative to all X-ray entries and entries of similar resolution. The OWAB column contains the minimum, median, 95th percentile and maximum values of the occupancy-weighted average B-factor per residue. The column labelled 'Q< 0.9' lists the number of (and percentage) of residues with an average occupancy less than 0.9.

Mol	Chain	Analysed	<RSRZ>	#RSRZ>2			OWAB(Å^2)	Q<0.9
1	A	288/289 (99%)	-0.48	0	100	100	16, 36, 66, 86	0
1	B	285/289 (98%)	-0.06	11 (3%)	37	33	30, 64, 96, 124	0
1	C	285/289 (98%)	-0.41	2 (0%)	84	86	16, 35, 64, 91	0
1	D	288/289 (99%)	-0.33	1 (0%)	91	92	18, 40, 68, 85	0
1	E	284/289 (98%)	-0.12	5 (1%)	65	64	31, 62, 106, 140	0
1	F	285/289 (98%)	-0.49	1 (0%)	90	91	13, 33, 60, 82	0
All	All	1715/1734 (98%)	-0.32	20 (1%)	75	77	13, 43, 90, 140	0

All (20) RSRZ outliers are listed below:

Mol	Chain	Res	Type	RSRZ
1	E	284	TRP	5.1
1	E	272	GLY	3.4
1	E	270	LYS	3.2
1	C	280	PRO	3.2
1	B	284	TRP	2.9
1	B	3	ALA	2.8
1	B	66	PHE	2.8
1	F	288	LEU	2.7
1	B	193	ASP	2.4
1	B	286	ALA	2.4
1	B	278	THR	2.4
1	B	154	LEU	2.2
1	B	274	ILE	2.2
1	D	249	THR	2.2
1	E	224	ILE	2.2
1	B	236	PHE	2.1
1	E	246	TYR	2.1
1	C	278	THR	2.1
1	B	155	GLU	2.1

Continued on next page...

Continued from previous page...

Mol	Chain	Res	Type	RSRZ
1	B	188	LEU	2.1

6.2 Non-standard residues in protein, DNA, RNA chains (i)

There are no non-standard protein/DNA/RNA residues in this entry.

6.3 Carbohydrates (i)

There are no carbohydrates in this entry.

6.4 Ligands (i)

In the following table, the Atoms column lists the number of modelled atoms in the group and the number defined in the chemical component dictionary. LLDF column lists the quality of electron density of the group with respect to its neighbouring residues in protein, DNA or RNA chains. The B-factors column lists the minimum, median, 95th percentile and maximum values of B factors of atoms in the group. The column labelled 'Q< 0.9' lists the number of atoms with occupancy less than 0.9.

Mol	Type	Chain	Res	Atoms	RSR	LLDF	B-factors(Å^2)	Q<0.9
3	EDO	F	1289	4/4	0.21	3.77	42,46,48,49	0
3	EDO	A	1289	4/4	0.17	2.16	34,35,35,38	0
3	EDO	D	1290	4/4	0.15	0.71	31,34,37,42	0
3	EDO	D	1289	4/4	0.14	0.51	39,39,40,43	0
2	ZN	F	1001	1/1	0.12	-0.21	30,30,30,30	0
2	ZN	D	1001	1/1	0.12	-0.48	31,31,31,31	0
2	ZN	B	1001	1/1	0.10	-0.81	48,48,48,48	0
2	ZN	C	1000	1/1	0.11	-0.96	39,39,39,39	0
2	ZN	F	1000	1/1	0.10	-1.27	33,33,33,33	0
2	ZN	E	1001	1/1	0.07	-1.28	54,54,54,54	0
2	ZN	E	1000	1/1	0.06	-1.78	55,55,55,55	0
2	ZN	D	1000	1/1	0.10	-2.27	37,37,37,37	0
2	ZN	C	1001	1/1	0.09	-2.36	31,31,31,31	0
2	ZN	A	1001	1/1	0.09	-2.69	30,30,30,30	0
2	ZN	B	1000	1/1	0.10	-2.70	50,50,50,50	0
2	ZN	A	1000	1/1	0.08	-3.60	28,28,28,28	0

6.5 Other polymers (i)

There are no such residues in this entry.
