# Supplementary material for: Identification of LACTB2, a metallo-β-lactamase protein, as a human mitochondrial endoribonuclease
Source: Nucleic Acids Res. 2016 Jan 29;44(4):1813–32. doi: 10.1093/nar/gkw050 (PMC4770246; doi:10.1093/nar/gkw050)
Supplement: SUPPLEMENTARY DATA [file supp_44_4_1813__index.html]

Identification of LACTB2, a metallo-β-lactamase protein, as a human mitochondrial endoribonuclease — SUPPLEMENTARY DATA 

# Identification of LACTB2, a metallo-β-lactamase protein, as a human mitochondrial endoribonuclease

## SUPPLEMENTARY DATA

- SUPPLEMENTARY DATA
- SUPPLEMENTARY DATA
